# Statin Use and Survival Among Men Receiving Androgen-Ablative Therapies for Advanced Prostate Cancer: A Systematic Review and Meta-analysis

**DOI:** 10.1001/jamanetworkopen.2022.42676

**Published:** 2022-11-30

**Authors:** Viranda H. Jayalath, Roderick Clark, Katherine Lajkosz, Rouhi Fazelzad, Neil E. Fleshner, Laurence H. Klotz, Robert J. Hamilton

**Affiliations:** 1 Division of Urology, Department of Surgery, University of Toronto, Toronto, Ontario, Canada; 2 Women’s College Research Institute, Toronto, Ontario, Canada; 3 Division of Surgical Oncology-Urology, Princess Margaret Cancer Center, Toronto, Ontario, Canada; 4 Library Services, University Health Network, Toronto, Ontario, Canada; 5 Division of Urology, Sunnybrook Health Sciences Centre, Toronto, Ontario, Canada

## Abstract

**Importance:**

Epidemiological evidence supports a role for statins in improving survival in advanced prostate cancer, particularly among men receiving androgen-ablative therapies.

**Objective:**

To study the association between statin use and survival among men with prostate cancer receiving androgen deprivation therapy (ADT) or androgen receptor axis–targeted therapies (ARATs).

**Data Sources:**

This systemic review and meta-analysis used sources from MEDLINE, EMBASE, Epub Ahead of Print, Cochrane Clinical Trials, Cochrane Systematic Reviews, and Web of Science from inception to September 6, 2022.

**Study Selection:**

Observational studies reporting associations of concurrent statin use and survival outcomes (in hazard ratios [HRs]).

**Data Extraction and Synthesis:**

Two authors independently abstracted all data. Summary estimates pooled multivariable HRs with 95% CIs using the generic inverse variance method with random-effects modeling. A priori specified subgroup and sensitivity analyses were undertaken, and heterogeneity, study quality, and publication bias were evaluated. Confidence in the evidence was assessed using the GRADE (Grading of Recommendations Assessment, Development, and Evaluation) approach.

**Main Outcomes and Measures:**

Overall mortality and prostate cancer–specific mortality (PCSM).

**Results:**

Twenty-five cohorts of 119 878 men (65 488 statin users [55%]) with more than 74 416 deaths were included. Concurrent statin use was associated with a 27% reduction in the risk of overall mortality (HR, 0.73 [95% CI, 0.66-0.82]; *I*^2^ = 83%) and a 35% reduction in the risk of PCSM (HR, 0.65 [95% CI, 0.58-0.73]; *I*^2^ = 74%), with substantial heterogeneity in both estimates. Subgroup analyses identified a PCSM advantage associated with statins for men receiving ARATs compared with ADT alone (HR, 0.40 [95% CI, 0.30-0.55] vs 0.68 [95% CI, 0.60-0.76]; *P* = .002 for difference). Confidence in the evidence was rated low for both outcomes.

**Conclusions and Relevance:**

The findings of this meta-analysis show that concurrent statin use was associated with reduced overall mortality and PCSM among men receiving androgen-ablative therapies for advanced prostate cancer. These findings are limited by the observational nature of the data and residual unexplained interstudy heterogeneity. Randomized clinical trials are warranted to validate these results.

## Key Points

**Question:**

Is statin use during androgen-ablative therapies (androgen deprivation or androgen receptor axis–targeted therapies) associated with reduced mortality among men with prostate cancer?

**Findings:**

In this systematic review and meta-analysis of 25 cohorts including 119 878 men, concurrent statin use was associated with a 27% reduced risk of overall mortality and a 35% reduced risk of prostate cancer–specific mortality.

**Meaning:**

These findings suggest that concurrent statin use may improve survival in men receiving androgen-ablative therapies for advanced prostate cancer; randomized clinical trials are warranted to confirm these findings.

## Teaser

This systematic review and meta-analysis quantifies the association between concurrent statin use and overall and prostate cancer−specific mortality among men undergoing androgen-ablative therapies for advanced prostate cancer.

## Introduction

Androgen deprivation therapy (ADT), whether through bilateral orchidectomy or luteinizing hormone–releasing hormone agonists or antagonists, is the standard of care for men with advanced prostate cancer.^1,2^ With recent level I evidence buttressing a substantial survival advantage, further androgen ablation through androgen receptor axis−targeted therapies (ARATs) has also been recommended for men with high-risk hormone-sensitive prostate cancer (HSPC) or castration-resistant prostate cancer (CRPC).^1,2^ Although nearly all patients initially respond to these androgen-ablative therapies, progression to castration-resistant disease remains an eventual certainty. With a median survival of 3 to 6 years from ADT initiation,^3^ there is an urgent need for therapies that can delay progression and improve survival in this population.

Statins, which are prescribed to lower cholesterol levels, have drawn recent attention as a potential adjunctive therapeutic in prostate cancer. Although statins appear to preferentially benefit later-stage disease, the subpopulation in which statins may be most efficacious remains unclear.^4^ One potential cohort includes men receiving castration therapy. Preclinical studies suggest that statins may work synergistically with androgen-ablative therapies to limit intratumoral steroidogenesis^5^ and inhibit adrenal androgen transport into prostate cancer cells.^6^ Observational data support this mechanism and suggest a survival advantage in men with CRPC. Indeed, statins appear to prolong the time to progression while using ADT and ARATs.^6,7^ A recent meta-analysis^8^ found statins to be associated with a modestly lower risk of biochemical recurrence; however, this association was nullified after excluding men receiving ADT. Several additional studies have since reported a protective association between statins and survival among men receiving androgen-ablative therapies.^9^

A comprehensive up-to-date synthesis of this association to aid clinical decision-making is lacking. Thus, we conducted a systematic review and meta-analysis to quantify the association between concurrent statin use and overall mortality and prostate cancer-specific mortality (PCSM) among men undergoing androgen-ablative therapies for advanced prostate cancer.

## Methods

This study was conducted in accordance with the *Cochrane Handbook for Systematic Reviews of Interventions*^10^ and reported in compliance with the Meta-analysis of Observational Studies in Epidemiology (MOOSE) guidelines. Our study protocol was registered (PROSPERO identifier: CRD42021237047). This study was exempt from research ethics board review as per University of Toronto guidelines on human research.^11^

### Literature Search

Using a librarian-guided comprehensive search strategy, MEDLINE, EMBASE, Epub Ahead of Print, Cochrane Clinical Trials, Cochrane Systematic Reveiws, and Web of Science were searched from inception to September 6, 2022 (eAppendix 1 in Supplement 1). A manual review of reference lists of included articles and previously published systematic reviews supplemented the database search.

### Study Selection and Data Extraction

Observational cohort studies reporting an association (as hazard ratios [HRs]) between concurrent statin use and survival outcomes (overall mortality or PCSM) in men undergoing androgen-ablative therapies for prostate cancer were eligible. To minimize influence of prevalent users, studies that evaluated statin exposure before initiation of castration therapy were excluded. Titles and abstracts of all studies were initially assessed by 1 author (V.H.J.) and a full-text review was undertaken by 2 independent authors (V.H.J. and R.C.). If multiple publications derived data from the same cohort, preference was given to the study that provided larger sample sizes, was available as full text, or reported more complete data for subgroup analyses. All discrepancies were resolved by discussion, and by consulting a senior author (R.J.H.) when necessary.

Two authors (V.H.J. and R.C.) independently extracted relevant demographic, exposure, outcome, and statistical data from eligible studies. Subpopulation-specific data (eg, chemotherapy vs no chemotherapy, abiraterone acetate vs enzalutamide, locally advanced vs metastatic cancer) were preferred, as opposed to pooled estimates, whenever possible. Individual study authors were contacted for additional data. Data on race and ethnicity were not specifically available in the published reports.

### Risk of Bias

The Newcastle-Ottawa Scale (NOS; 9-point scale) quantified risk of bias across the domains of cohort selection (4 points), comparability of design or analysis (2 points), and adequacy of outcome measures (3 points).^12^ A study was considered high quality with a low risk of bias if the NOS score was greater than 6 points.

### Quality of Evidence

The GRADE (Grading of Recommendations Assessment, Development, and Evaluation) approach was used to evaluate the quality of evidence for the primary outcomes and categorized as high, moderate, low, or very low.^13^ Two authors (V.H.J. and R.C.) without conflicts of interest related to this study reviewed the synthesized evidence and downgraded its certainty based on study design, risk of bias, inconsistency, indirectness, and imprecision.

### Statistical Analysis

#### Data Synthesis

The 2 primary outcomes consisted of the association between concurrent statin use and overall mortality and PCSM. For each, we meta-analyzed individual HRs with corresponding 95% CIs from the most adjusted multivariable model, using the generic inverse variance approach with random-effects modeling. Pooled summary estimates were reported as HRs with 95% CIs.

#### Heterogeneity

Interstudy heterogeneity was determined using Cochran *Q* test (significant at α < .10) and quantified using the *I*^2^ statistics (0%-100%, where >50% was considered substantial). Subgroup analysis by meta-regression was undertaken if heterogeneity was significant and at least 10 cohorts were available. A priori subgroups included median age (≤70 vs >70 years), baseline metastasis (nonmetastatic vs mixed vs metastatic), hormone sensitivity (HSPC vs CRPC), type of androgen-ablative therapy (ADT vs ARAT), type of primary treatment (ADT alone vs other), adjustment for cardiovascular covariates (yes vs no), and NOS-based study quality (high vs low).

#### Sensitivity Analysis

We performed 4 a priori specified sensitivity analyses. We first removed each cohort individually from the meta-analysis and recalculated the summary estimate (leave-one-out approach). Second, we removed all studies that were available only in the form of abstracts. Third, to evaluate for any undue influence of small studies on the summary estimate, we repeated the meta-analysis using fixed-effects modeling. Last, owing to the vulnerability of cohorts of statin users to immortal time bias because patients have to survive long enough to start statin therapy,^14^ we performed a sensitivity analysis by removing all studies potentially susceptible to immortal time bias. A cohort was considered susceptible if (1) no time-dependent analysis was performed or (2) information regarding the timing of statin exposure was not established or available.

#### Dose-Response Analysis

A dose-response analysis was planned a priori; however, because insufficient data were available for meta-analysis, only a systematic review was reported. Similarly, analyses of statin class (hydrophilic vs lipophilic) were precluded by limited data availability.

#### Publication Bias

Publication bias was assessed by visually inspecting funnel plots for asymmetry. Results were formally tested using the Egger test (*P* < .10 indicated significant publication bias).

#### Software

Primary meta-analyses were performed on Review Manager, version 5.4 (The Nordic Cochrane Centre, The Cochrane Collaboration). All other analyses were undertaken on R, version 3.6.1 (R Project for Statistical Computing). GRADE assessments were performed on GRADEpro (McMaster University and Evidence Prime Inc).

## Results

### Literature Flow

Systematic literature review identified 5190 records, of which 54 reports were reviewed in full. After completing full-text reviews (interrater agreement, 93%; Cohen κ = 0.86), we identified 19 publications^15,16,17,18,19,20,21,22,23,24,25,26,27,28,29,30,31,32,33^ reporting data from 25 cohorts of 119 878 men (65 488 [55%] statin users) to be included (eFigure 1 in Supplement 1). Overall mortality was reported in 19 cohorts (16 publications^15,16,17,18,19,20,21,22,25,27,28,29,30,31,32,33^), whereas PCSM was reported in 14 cohorts (10 publications^15,17,19,21,23,24,26,30,32,33^).

### Cohort Characteristics

Individual cohort characteristics are summarized in the Table and eTable 1 in Supplement 1. Included studies were published between 2014 and 2021 and collected data between 1992 and 2018. Data were available from multinational cohorts in 7 studies,^17,18,19,20,21,27,29^ multicenter cohorts in 7 studies,^15,23,26,30,31,32,33^ and single-institution experiences in 5 studies.^16,22,24,25,28^ The median age across study participants ranged from 62 to 75 years. Most cohorts consisted of men with newly diagnosed hormone-sensitive disease (n = 16). Seventeen cohorts^15,17,21,23,24,26,27,28,29,30,31,32,33^ had ADT exposure as the primary androgen-ablative therapy, whereas 7 cohorts^16,18,19,20,22,25^ had ARAT exposure (5 for abiraterone alone, 1 for enzalutamide alone, and 1 for both). Eight cohorts^17,20,23,28,30,31^ included chemotherapy-naive patients, whereas 5 cohorts^19,20,27,29^ evaluated patients post chemotherapy, and 8 cohorts^15,16,18,21,24,26,32,33^ evaluated both.

**Table.** Characteristics of Cohorts Included in Meta-analysis

Source	Cohort^a^	Study years	Study size	Age, y^b^	Stage	Chemotherapy	Castration	Statin users, No. (%)^c^	Outcome	Survival, median (IQR), mo	OM events, No. (%)	Study quality^d^
Anderson-Carter et al,^15^ 2019	US (VA cohort)	2000-2016	87 347	Range, 73-76	M0 HSPC	Mixed	ADT	53 360 (61)	OM and PCSM	Users: 78 (41-120); nonusers: 48 (24-88)	Users: 37 721 (71); nonusers: 29 547 (87)	High
Boegemann et al,^16^ 2016	Germany (single center)	2010-2015	108	70	M1 CRPC	Mixed	Abiraterone	21 (19)	OM	Users: 14 (9.8-18); nonusers: 18 (14-22)	Users: 15 (71); nonusers: 60 (69)	High
Calais Da Silva et al,^17^ 2014^e^	Multinational (SEUG-9901 trial)	1999-2012	339	Range, 70-74	M0 to M1 HSPC	No	ADT	147 (43)	OM and PCSM	NA	Users: 37 (25); nonusers: 76 (40)	Low
Di Lorenzo et al,^18^ 2018	Multinational	2011-2016	187	Range, 71-72	M1 CRPC	Mixed	Abiraterone	71 (38)	OM	Users: 22 (19-25); nonusers: 15 (13-17)	Users: 57 (80); nonusers: 96 (84)	Low
Gordon et al,^19^ 2018	Multinational (STABEN cohort)	2011-2016	598	72	M1 CRPC	Yes	Abiraterone and enzalutamide	199 (33)	OM and PCSM	Users: 21 (18-23); nonusers: 13 (11-15)	Users: 166 (83); nonusers: 347 (87)	High
Hamilton et al,^20^ 2014^e^	Multinational (COU301 and COU302 trials)	2008-2011	COU301: 797; COU302: 546	COU301: 69; COU302: 71	M1 CRPC	COU301: yes; COU302: no	Abiraterone	COU301: 236 (30); COU302: 229 (42)	OM	NA	NA	Low
Hamilton et al,^21^ 2021	Multinational (PR-7 trial)	1999-2005	1364	Range, 73-75	M0 HSPC	Mixed	ADT	585 (43)	OM and PCSM	Users: 126 (120-132); nonusers: 89 (84-96)	All: 513 (38)	High
Henriquez Lopez et al,^22^ 2019^e^	Spain (single center)	2009-2018	64	Range, 68-70	M1 CRPC	NA	Abiraterone and enzalutamide	32 (50)	OM	Users: 43 (36-50); nonusers: 30 (23-36)	NA	Low
Joentausta et al,^23^ 2019	Finland (multicenter)	1996-2014	2926	62	M0 HSPC	No	ADT	1335 (46)	PCSM	NA	NA	High
Jung et al,^24^ 2015	Korea (single center)	1997-2013	171	67	M1 HSPC	Mixed	ADT	46 (27)	PCSM	Users: 58; nonusers: 45	NA	High
Lai et al,^25^ 2020^e^	US (single center)	2011-2017	156	NA	M1 HSPC	NA	Abiraterone	NA	OM	NA	NA	Low
Larsen et al,^26^ 2017	Denmark (multicenter)	1998-2013	8269	70	M0 and M1 HSPC	Mixed	ADT	ADT alone: 1370 (NA); ADTplus RT: 1117 (NA)	PCSM	NA	NA	High
Lorente et al,^27^ 2018^e^	Multinational (TROPIC trial)	2007-2008	755	Range, 67-68	M1 CRPC	Yes	ADT	138 (18)	OM	Users: 16; nonusers: 13	All: 502 (66)	High
Mikkelsen et al,^28^ 2017	Denmark (single center)	2007-2013	537	Range, 74-75	M0 and M1 HSPC	No	ADT	141 (26)	OM	NA	NA	High
Niraula et al,^29^ 2013	Multinational (TAX 327 trial)	2000-2003	1006	68	M1 CRPC	Yes	ADT	82 (8)	OM	Users: 17 (15-23); nonusers: 17 (16-18)	All: 557 (55)	High
Peltomaa et al,^30^ 2021	Finland (FinRSPC trial)	1996-2015	4428	Range, 70-71	M0 HSPC	No	ADT	2544 (57)	OM and PCSM	Users: 82; nonusers: 71	Users: 918 (36); nonusers: 1009 (54)	High
Siddiqui et al,^31^ 2019^e^	USA (VANCHCS cohort)	1992-2016	110	NA	M1 HSPC	No	ADT	27 (25)	OM	NA	NA	Low
Tan et al,^32^ 2020	US (SEER cohort)	2008-2012	4421	74	M0 and M1 HSPC	Mixed	ADT	1957 (44)	OM and PCSM	NA	NA	High
Wu et al,^33^ 2019	Taiwan (NHIRD cohort)	2008-2014	5749	73	M0 HSPC andM1 HSPC	Mixed	ADT	M0: 750 (40); M1: 1101 (35)	OM and PCSM	NA	Users (M0): 154 (21); nonusers (M0): 271 (24); users (M1): 630 (57); nonusers (M1): 1204 (59)	High

Abbreviations: ADT, androgen-deprivation therapy; CRPC, castration-resistant prostate cancer; FinRSPC, Finnish Randomized Study of Screening for Prostate Cancer; HSPC, hormone-sensitive prostate cancer; NA, not available; OM, overall mortality; PCSM, prostate cancer–specific mortality; RT, radiation therapy.

^a^ All cohorts were retrospective cohorts in design.

^b^ Median age was preferred where available, otherwise range or mean age was used.

^c^ All users had concomitant statin and androgen-ablative therapy use; any statin type/dose was permitted.

^d^ Evaluated using the Newcastle-Ottawa Scale (0-9 points), where studies that achieved more than 6 points were considered high quality. A complete breakdown of scoring for each study is reported in eTable 3 in Supplement 1.

^e^ Data available only as abstract.

Cohorts ascertained statin use from prescription databases,^15,23,26,30,32,33^ patient interviews,^16,17,20,21,27,29^ and retrospective medical record reviews^18,19,24,28,31^; methods of exposure assessment were not reported in 2 cohorts.^22,25^ All studies evaluated statin exposure occurring concurrently alongside androgen-ablative therapies.

All but 1 cohort^20^ reported multivariable-adjusted models; 2 cohorts^15,32^ adjusted for propensity scores (eTable 2 in Supplement 1). Only 6 studies^18,19,22,25,28,32^ adjusted for more than 3 pathological variables. Relevant prognostic covariates adjusted for included prostate-specific antigen levels in 8 studies,^15,18,19,21,22,25,27,28^ Gleason score in 8 studies,^15,18,19,24,25,26,28,33^ metastatic burden in 7 studies,^16,17,18,19,25,27,28^ and alkaline phosphatase in 4 studies.^18,19,22,27^ Seventeen cohorts in 12 studies^15,19,23,24,26,27,28,29,30,31,32,33^adjusted for at least 1 variable associated with cardiovascular mortality.

### Overall Mortality

**Figure 1.**
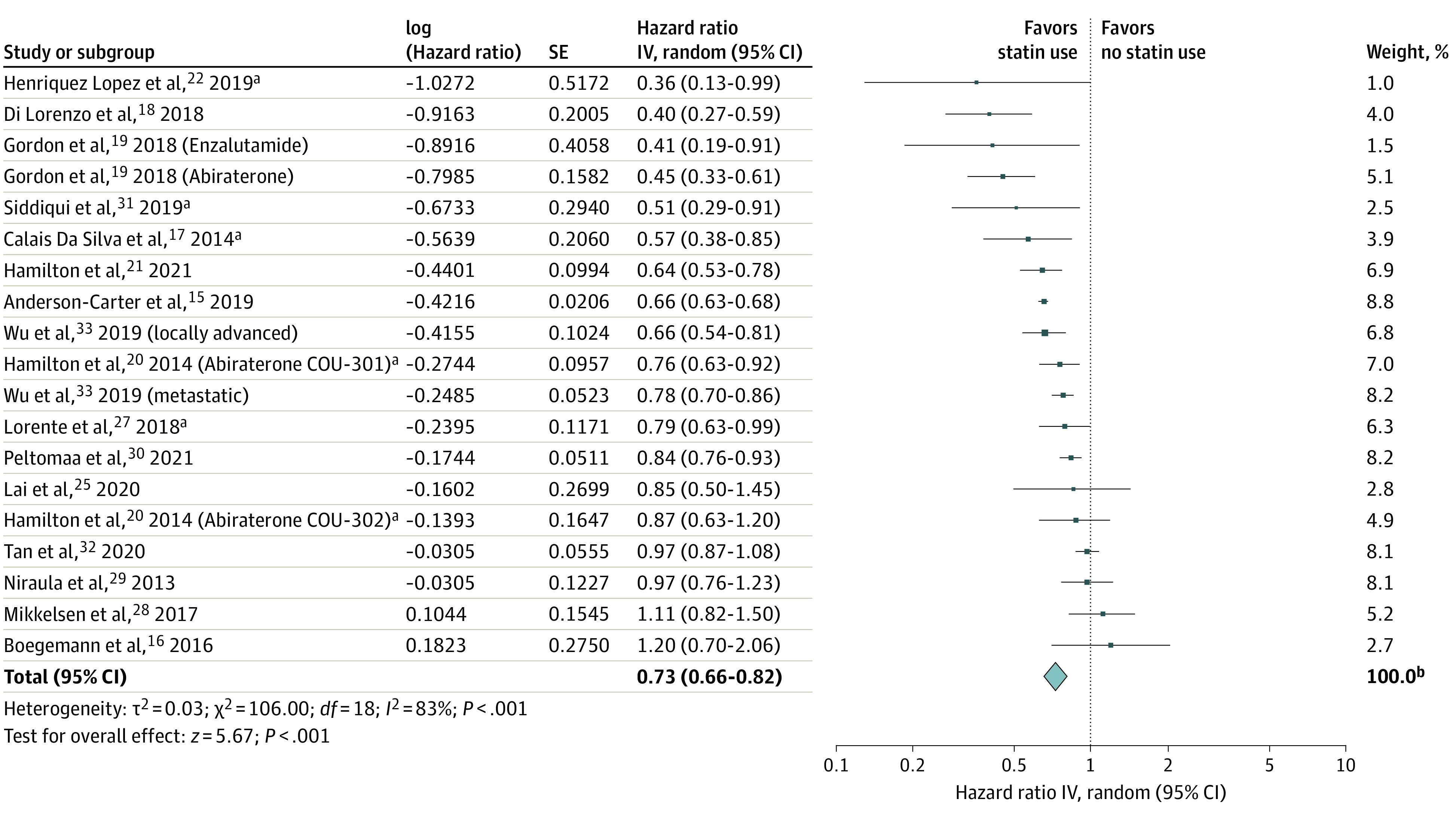
Forest Plot of the Association Between Postdiagnostic Statin Use and Overall Mortality Among Men Receiving Androgen-Ablative Therapies for Advanced Prostate Cancer Includes 19 cohorts and 108 512 men. The diamond represents the pooled estimate, derived from generic inverse variance (IV) random-effects modeling. Each square represents individual study estimates; the size of each square corresponds to the weight attributed to each cohort in the overall summary estimate. The horizontal lines represent 95% CIs. Interstudy heterogeneity was assessed using the Cochran *Q* statistic and quantified with the *I*^2^ statistic (>50% is considered substantial). ^a^Data available only in the form of an abstract. ^b^Owing to rounding, individual values sum to greater than 100%.

Nineteen retrospective cohorts of 108 512 men (61 950 [57%] statin users) with more than 73 885 mortality events (deaths reported for 12 cohorts) were included in a meta-analsis.^15,16,17,18,19,20,21,22,25,27,28,29,30,31,32,33^ Concurrent statin use was associated with a 27% reduction in the risk of overall mortality (HR, 0.73 [95% CI, 0.66-0.82]) in random-effects meta-analysis (Figure 1), with significant heterogeneity between cohorts (*I*^2^ = 83%; *P* < .001).

#### Subgroup and Sensitivity Analyses

**Figure 2.**
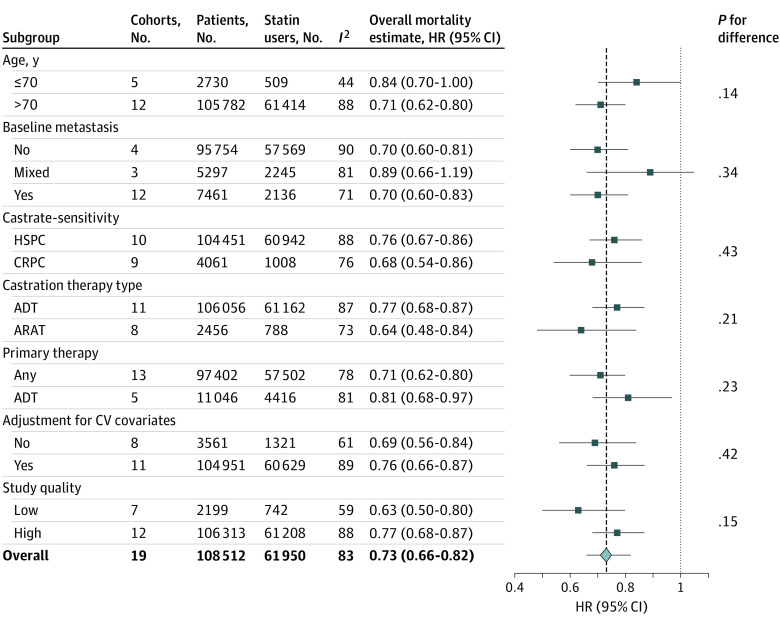
A Priori Subgroup Analyses Evaluating the Association Between Postdiagnostic Statin Use and Risk of Overall Mortality Among Men Receiving Androgen-Ablative Therapies for Advanced Prostate Cancer Includes 19 cohorts and 108 512 men. The diamond represents the pooled estimate, derived from generic inverse variance random-effects modeling. Each square represents the respective subgroup pooled estimate. The horizontal lines represent 95% CIs. Interstudy heterogeneity was assessed using the Cochran *Q* statistic and quantified with the *I*^2^ statistic (>50% is considered substantial). ADT indicates androgen deprivation therapy; ARAT, androgen receptor axis–targeted therapy; CRPC, castration-resistant prostate cancer; CV, cardiovascular; HR, hazard ratio; HSPC, hormone-sensitive prostate cancer.

None of the a priori subgroups modified the observed association or the interstudy heterogeneity (Figure 2). The directionality and the significance of the estimate were not altered by systematic removal of individual cohorts from the pooled analysis (range of HRs, 0.72-0.75) nor by removing 6 cohorts available only as abstracts (HR, 0.74). Recalculating the meta-analysis using a fixed-effect model did not modify the findings (HR, 0.71 [95% CI, 0.69-0.74]; *I*^2^ = 83%), although 58% of the observed association was weighted on 1 cohort. Removal of this cohort diminished the magnitude of the association, although the pooled estimate remained significant (HR, 0.80 [95% CI, 0.76-0.84]; *I*^2^ = 74%). Last, removing cohorts susceptible to immortal time bias^18,22,24,25,28,31^ did not significantly change the overall association (HR, 0.75 [95% CI, 0.67-0.84]).

#### Dose-Response Assessments

Four studies^15,19,30,33^ assessed a statin dose-response association for overall mortality (eTable 3 in Supplement 1). Three studies^15,30,33^ evaluated statin defined daily dose categories and supported a dose-response association, with HRs ranging from 0.61 (95% CI, 0.51-0.71) to 0.90 (95% CI, 0.82-0.99) in the highest exposure categories.^3^ One study^19^ used a continuous model per milligram of statin dose equivalent and failed to identify a significant dose-response association (HR, 1.00 [95% CI, 0.99-1.01]).

#### Publication Bias, Risk of Bias (NOS), and Quality of Evidence (GRADE)

There was no evidence of publication bias in visual inspection of funnel plots (eFigure 2 in Supplement 1), nor formal evaluation by Egger test (*P* = .57). Study quality was high (NOS score, >6) for 13 of 19 studies^15,16,19,21,23,24,26,27,28,29,30,32,33^ (eTable 4 in Supplement 1). Among the 6 reports considered low quality, 5 were available only as abstracts,^17,20,22,25,31^ and points were lost for inadequate descriptions of cohort derivation, ascertainment of exposure, and assessment of outcome. Confidence in the estimate of the association between statins and overall mortality was low, owing to the observational nature of the included studies and significant residual unexplained heterogeneity (eAppendix 2 in Supplement 1).

### Prostate Cancer–Specific Mortality

**Figure 3.**
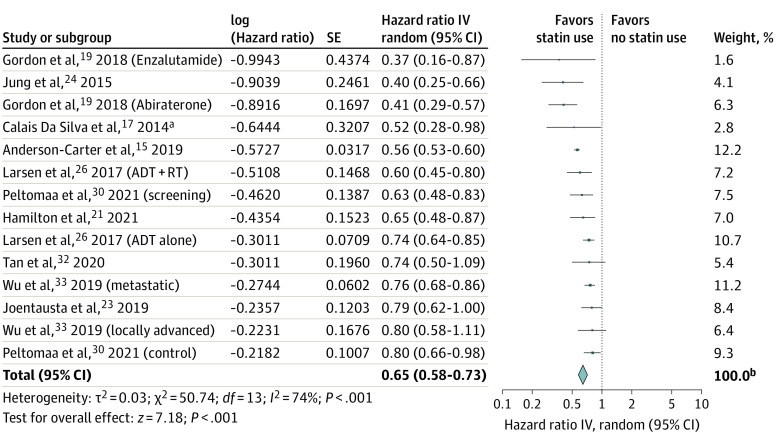
Forest Plot of the Association Between Postdiagnostic Statin Use and Prostate Cancer–Specific Mortality Among Men Receiving Androgen-Ablative Therapies for Advanced Prostate Cancer Includes 14 cohorts and 115 612 men. The diamond represents the pooled estimate, derived from generic inverse variance (IV) random-effects modeling. Each square represents individual study estimates; the size of each square corresponds to the weight attributed to each cohort in the overall summary estimate. The horizontal lines represent 95% CIs. Interstudy heterogeneity was assessed using the Cochran *Q* statistic and quantified with the *I*^2^ statistic (>50% is considered substantial). ADT indicates androgen deprivation therapy; RT, radiation therapy. ^a^Data available only in the form of an abstract. ^b^Owing to rounding, individual values sum to greater than 100%.

Fourteen retrospective cohorts of 115 612 men (64 511 [56%] statin users) with more than 12 408 mortality events (deaths reported for 11 cohorts) were included in a meta-analysis.^15,17,19,21,23,24,26,30,32,33^ Concurrent statin use was associated with a 35% reduction in the risk of PCSM (HR, 0.65 [95% CI, 0.58-0.73]) in random-effects meta-analysis (Figure 3), with significant heterogeneity between cohorts (*I*^2^ = 74%; *P* < .001).

#### Subgroup and Sensitivity Analyses

**Figure 4.**
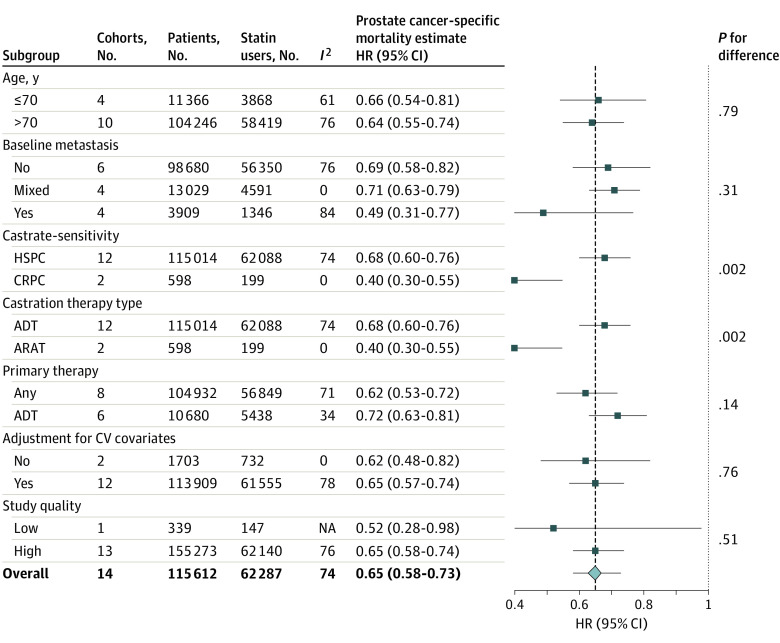
A Priori Subgroup Analyses Evaluating the Association Between Postdiagnostic Statin Use and Risk of Prostate Cancer–Specific Mortality Among Men Receiving Androgen-Ablative Therapies Includes 14 cohorts and 115 612 men. The diamond represents the pooled estimate, derived from generic inverse variance random-effects modeling. Each square represents the respective subgroup pooled estimate. The horizontal lines represent 95% CIs. Interstudy heterogeneity was assessed using the Cochran *Q* statistic and quantified with the *I*^2^ statistic (>50% is considered substantial). ADT indicates androgen deprivation therapy; ARAT, androgen receptor axis–targeted therapy; CRPC, castration-resistant prostate cancer; CV, cardiovascular; HR, hazard ratio; HSPC, hormone-sensitive prostate cancer; NA, not applicable.

Of the evaluated a priori subgroups, castration sensitivity (HSPC vs CRPC) and androgen-ablative therapy type (ADT vs ARAT), both with identical cohort distributions across subgroups, significantly modified the pooled association (Figure 4). Statin exposure was associated with a significantly lower risk of PCSM among men with CRPC or those receiving ARATs (2 cohorts^19^ with 589 patients) compared with men with HSPC or those receiving ADT (12 cohorts^15,17,21,23,24,26,30,32,33^ with 115 014 patients) (HRs, 0.40 [95% CI, 0.30-0.55] vs 0.68 [95% CI, 0.60-0.76]; *P* = .002 for difference). Neither the directionality nor the significance of the observed association were altered by systematic removal of individual cohorts from the pooled analysis (range of HRs, 0.64-0.67) or by removing 1 cohort available only as an abstract (HR, 0.65). Recalculating the meta-analysis using a fixed-effect model did not suggest a strong influence of small studies (HR, 0.63 [95% CI, 0.60-0.66]; *I*^2^ = 74%), although 52% of the observed associated was weighted on 1 cohort. Removal of this cohort from the fixed-effects model reduced the magnitude of the association, although the pooled estimate remained significant (HR, 0.71 [95% CI, 0.66-0.76]; *I*^2^ = 53%). Last, removing cohorts susceptible to immortal time bias^18,22,24,25,28,31^ did not significantly alter the overall association (HR, 0.66 [95% CI, 0.59-0.75]).

#### Dose-Response Evaluation

Four cohorts^15,19,30,33^ assessed a statin dose-response association for PCSM (eTable 3 in Supplement 1). Three cohorts^15,30,33^ evaluated statin defined daily dose categories and supported a dose-response association, with HRs ranging from 0.58 (95% CI, 0.44-0.76) to 0.74 (95% CI, 0.59-0.93) in the highest categories. One cohort^19^ used a continuous model per milligram of statin dose equivalent and failed to identify a significant dose-response association (HR, 1.00 [95% CI, 0.99-1.01]).

#### Publication Bias, Risk of Bias (NOS), and Quality of Evidence (GRADE)

There was no evidence of publication bias in visual inspection of funnel plots (eFigure 3 in Supplement 1) nor the formal evaluation by Egger test (*P* = .64). Study quality was high (NOS score >6) for 13 of 14 cohorts^15,19,21,23,24,26,30,32,33^ (eTable 4 in Supplement 1). The 1 low-quality study^17^ was available only as an abstract, with points lost for inadequate descriptions of outcome measures. Confidence in the estimate of the statin-PCSM association was low, primarily due to the observational nature of the included studies and significant residual unexplained heterogeneity (eAppendix 2 in Supplement 1).

## Discussion

This systematic review and meta-analysis studied the association between statin use and survival outcomes in men using androgen-ablative therapies for advanced prostate cancer. In pooling data from 25 cohorts and 119 878 men, we identified an association of statin use with a 27% overall mortality and 35% PCSM benefit, albeit with significant interstudy heterogeneity. Subgroup analyses suggested a preferential advantage for men receiving ARATs. These findings were not driven by any single cohort, withstood robust sensitivity analyses, and were free of publication bias.

### Clinical Context

There is great enthusiasm for the repurposing of common medications in the adjunctive treatment of prostate cancer. Although numerous drugs have been considered, statins stand out as an ideal chemopreventive candidate owing to low cost, well-established cardiovascular benefit, oral formulation, and a favorable adverse effects profile.^34^ Which subpopulation with prostate cancer may benefit most from statins remains unclear.

The literature supports a protective association of statins in preventing high-grade disease, with little to no association with low-grade disease.^35,36^ Similarly, statins do not appear to modify the risk of prostate cancer progression for men with low-risk disease undergoing active surveillance.^37^ Meta-analyses studying progression after definitive therapy (radical prostatectomy or radiation therapy)^8,38^ suggest a modest 10% to 12% risk reduction in biochemical recurrence with statins. This benefit, however, was rendered null after men receiving ADT were excluded from analyses.

The most consistent evidence of an association between statins and prostate cancer comes from studies of survival outcomes. Two meta-analyses^8,9^ noted a survival advantage of 25% to 35% among statin users before and after diagnosis, although subpopulation-specific assessments were not reported. Interestingly, a more recent cohort study^39^ identified a 20% lower risk of PCSM among men with postdiagnostic use of statins, although subgroup analyses revealed this risk reduction to be limited to men receiving hormonal therapy, with no advantage for men after surveillance, radical prostatectomy, or radiation therapy.

Our study represents, to our knowledge, the most comprehensive summary to date on the associations between statin therapy, androgen-ablative therapy, and prostate cancer. We observed a consistent overall and prostate cancer–specific survival advantage for statin users undergoing androgen-ablative therapies, independent of patient age, baseline metastasis status, prior use of chemotherapy, or primary treatment type. For overall mortality, the observed benefit was independent of hormone sensitivity status and type of androgen-ablative therapy. Conversely for PCSM, there was an incremental advantage of statins for men receiving ARATs compared with men receiving ADT. Although a dose-response analysis was planned as part of this study, the heterogeneity of available data precluded formal statistical assessments. Of the 4 cohorts that investigated a dose-response association, 3 suggested greater survival with increasing cumulative statin dose,^15,30,33^ whereas 1 found no association.^19^ It is unclear why no association was noted in the study by Gordon et al,^19^ although the findings may relate to the dose classification used (continuous per-milligram simvastatin dose^19^ vs defined daily dose categories^15,30,33^) or the type of background androgen-ablative therapy (ARAT^19^ vs ADT^15,30,33^). Increasing duration of postdiagnostic statin use also appears to improve prostate cancer biomarkers in clinical trials,^40^ and studies of statins and prostate cancer survival among all men support a dose-response association.^26,41^ Taken together, there likely exists a dose-response association between statins and survival in advanced prostate cancer, although a specific dose cannot be recommended based on the available data. Considering that all cohorts evaluated men receiving statins for nononcologic indications, it stands to reason that commonly used moderate to high statin doses are likely sufficient to elicit the observed survival benefits.

### Potential Mechanisms

Several pathways may explain the observed association. Statins may inhibit inflammation, angiogenesis, cell proliferation, migration, adhesion, invasion, and promotion of apoptosis by disruption of cellular communication mechanisms.^4^

In addition, a synergistic effect among statins, reduction of cholesterol levels, and androgen-ablative therapies may play a role. Cholesterol is a precursor for androgen biosynthesis. In the castration setting, testicular androgens diminish, and prostate cancer relies on adrenal androgens, namely dehydroepiandrosterone sulfate (DHEAS), and intratumoral de novo steroidogenesis to drive androgen-dependent tumor progression.^42^ Indeed, lowering circulating cholesterol levels has been reported to decrease intraprostatic androgen levels and slow proliferation of prostate cancer.^5,43^ Moreover, statins and DHEAS enter prostate cancer cells via the same membrane transporter, *SLCO2B1*; statins competitively inhibit entry of DHEAS into prostate cancer cells, decrease available intratumoral androgen pools, and slow tumor proliferation.^6^ Reinforcing this mechanism at the population level, statin users were found to have a nearly 10-month longer time-to-progression while receiving ADT.^6^ Statins may also affect the ARAT, abiraterone, in a similar fashion. Abiraterone inhibits *CYP17A*, blocking the conversion of pregnenolone to DHEAS precursors, decreasing adrenal androgen biosynthesis.^44^ Abiraterone also enters prostate cancer cells through *SLCO2B1* to elicit intraprostatic antineoplastic effects.^45^ Thus, statins and abiraterone may work together to lower circulating and intraprostatic DHEAS levels. Observational data buttress this pathway, reporting a nearly 5-month longer time to progression while statin users were receiving abiraterone.^7^

Last, it is established that androgen-ablative therapies increase cardiometabolic risk by promoting visceral obesity, dyslipidemia, and dysglycemia.^46^ Statins may work to negate some of the adverse cardiometabolic effects of androgen-ablative therapies, contributing to a survival benefit.

### Limitations

Several limitations warrant discussion. First, the observational nature of these data limits causal inferences and raises the possibility of residual confounding from unadjusted prognostic factors. It is particularly noteworthy that serum cholesterol levels were not adjusted for in any included studies. Serum cholesterol levels appear to correlate with levels of prostate-specific antigen^47,48^ and may in part drive the statin–prostate cancer association,^49,50^ potentially contributing to residual confounding of these findings. Second, the retrospective nature of exposure assessment leading to uncaptured discontinuation of or nonadherence to statin therapy may limit the accuracy of the primary outcomes. Third, there remained unexplained interstudy heterogeneity in both primary outcomes. It is worth noting, however, that although variability remained considerable, the differences were between small and large protective associations; in other words, these findings appear to accurately support a statin benefit, although they may be restricted by imprecision.^51^ Fourth, possible influences of frequently encountered biases in studies investigating drug repurposing, namely immortal time bias and selection bias, must not be overlooked.^14,52^ Six cohorts included in this meta-analysis were susceptible to immortal time bias; however, a sensitivity analysis excluding these cohorts did not alter the primary results, suggesting minimal influence of immortal time. Although the influence of selection bias was not formally investigated, we restricted our analysis to statin users concurrently receiving androgen-ablative therapies, excluding cohorts with prediagnostic users to mitigate any overt effects of prevalent users. In our study, cohorts that analyzed prediagnostic users reported similar or worse survival outcomes, compared with new postdiagnostic users^30,33^; thus, it is unlikely that selection bias dramatically affected the observed association.

## Conclusions

The notion of incorporating a readily available medication with an established cardiovascular benefit and favorable toxicity profile, such as statins, in the treatment of prostate cancer is exciting. In this systematic review and meta-analysis of observational studies, we identified a significant overall mortality and PCSM advantage of concurrent statin use for men receiving androgen-ablative therapies.

These results contribute to our understanding of the associations between prostate cancer, the androgen axis, and statins and provide high-quality evidence supporting a chemopreventive role for statins in prostate cancer. However, the use of retrospective data and unexplained heterogeneity lower our confidence in directly incorporating these findings into clinical practice. In seeking the optimal setting for statins to succeed in treating prostate cancer, work remains to delineate an optimal statin dose and class. The time is ripe for well-designed, randomized clinical trials to evaluate the effect of statins on prostate cancer survival.

## Notes

Supplement 1. **eFigure 1.** PRISMA Diagram **eFigure 2.** Funnel Plot of Hazard Ratios (HR) Evaluating the Association Between Statin Use and Overall Mortality Among Men With Androgen-Ablative Therapies for Advanced Prostate Cancer **eFigure 3.** Funnel Plot of Hazard Ratios (HR) Evaluating the Association Between Statin Use and Overall Mortality Among Men With Androgen-Ablative Therapies for Advanced Prostate Cancer **eTable 1.** Additional Characteristics of Cohorts Included in Meta-analyses **eTable 2.** Multivariable Models for Each Study Included in Meta-analyses **eTable 3.** Dose-Response Association Between Postdiagnostic Statin Use and Survival Outcomes Among Men on Androgen-Ablative Therapies for Advanced Prostate Cancer **eTable 4.** Assessment of Study Quality for Cohorts Included in Meta-analysis Using Newcastle-Ottawa Scale
eReferences
**eAppendix 1.** Databases and Search Strategy Used **eAppendix 2.** GRADE Assessment of Quality of Evidence

Supplement 2.
Data Sharing Statement
