# Supplementary material for: Statin Use and Survival Among Men Receiving Androgen-Ablative Therapies for Advanced Prostate Cancer: A Systematic Review and Meta-analysis
Source: JAMA Netw Open. 2022 Nov 30;5(11):e2242676. doi: 10.1001/jamanetworkopen.2022.42676 (PMC9713611; doi:10.1001/jamanetworkopen.2022.42676)
Supplement: Supplement 1. — eFigure 1. PRISMA Diagram eFigure 2. Funnel Plot of Hazard Ratios (HR) Evaluating the Association Between Statin Use and Overall Mortality Among Men With Androgen-Ablative Therapies for Advanced Prostate Cancer eFigure 3. Funnel Plot of Hazard Ratios (HR) Evaluating the Association Between Statin Use and Overall Mortality Among Men With Androgen-Ablative Therapies for Advanced Prostate Cancer eTable 1. Additional Characteristics of Cohorts Included in Meta-analyses eTable 2. Multivariable Models for Each Study Included in Meta-analyses eTable 3. Dose-Response Association Between Postdiagnostic Statin Use and Survival Outcomes Among Men on Androgen-Ablative Therapies for Advanced Prostate Cancer eTable 4. Assessment of Study Quality for Cohorts Included in Meta-analysis Using Newcastle-Ottawa Scale eReferences eAppendix 1. Databases and Search Strategy Used eAppendix 2. GRADE Assessment of Quality of Evidence [file jamanetwopen-e2242676-s001.pdf]

Supplementary Online Content

Jayalath VH, Clark R, Lajkosz K, et al. Statin use and survival among men receiving androgen-ablative therapies for advanced prostate cancer: a systematic review and meta-analysis. *JAMA Netw Open*. 2022;5(11):e2242676. doi:10.1001/jamanetworkopen.2022.42676

eFigure 1. PRISMA Diagram
eFigure 2. Funnel Plot of Hazard Ratios (HR) Evaluating the Association Between Statin Use and Overall Mortality Among Men With Androgen-Ablative Therapies for Advanced Prostate Cancer
eFigure 3. Funnel Plot of Hazard Ratios (HR) Evaluating the Association Between Statin Use and Overall Mortality Among Men With Androgen-Ablative Therapies for Advanced Prostate Cancer
eTable 1. Additional Characteristics of Cohorts Included in Meta-analysis
eTable 2. Multivariable Models for Each Study Included in Meta-analyses
eTable 3. Dose-Response Association Between Postdiagnostic Statin Use and Survival Outcomes Among Men on Androgen-Ablative Therapies for Advanced Prostate Cancer
eTable 4. Assessment of Study Quality for Cohorts Included in Meta-analysis Using Newcastle-Ottawa Scale
eReferences
eAppendix 1. Databases and Search Strategy Used
eAppendix 2. GRADE Assessment of Quality of Evidence

This supplementary material has been provided by the authors to give readers additional information about their work.

© 2022 Jayalath VH et al. *JAMA Network Open*.

eFigure 1. PRISMA Diagram

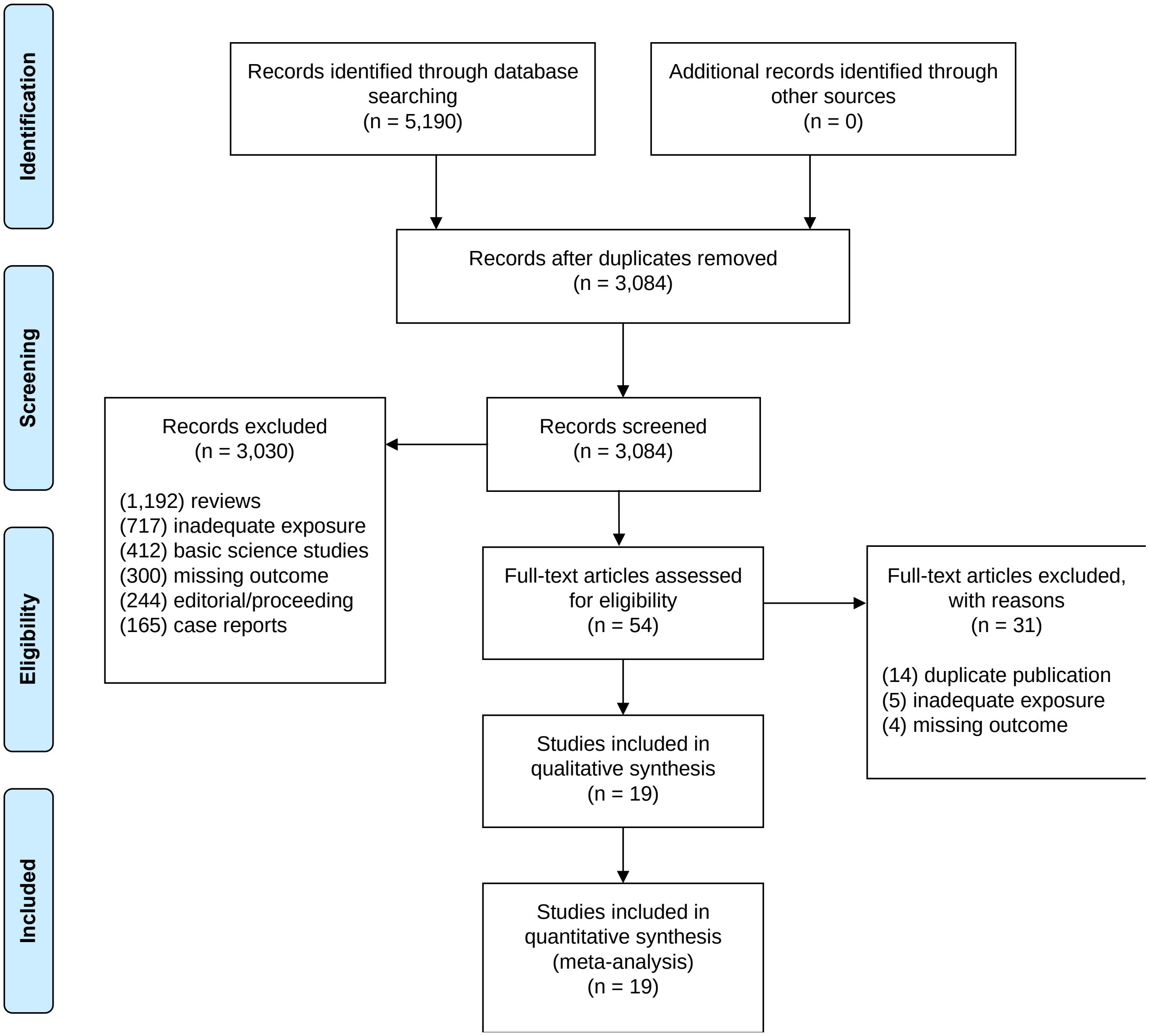

© 2022 Jayalath VH et al. *JAMA Network Open*.

eFigure 2. Funnel Plot of Hazard Ratios (HR) Evaluating the Association Between Statin Use and Overall Mortality Among Men With Androgen-Ablative Therapies for Advanced Prostate Cancer

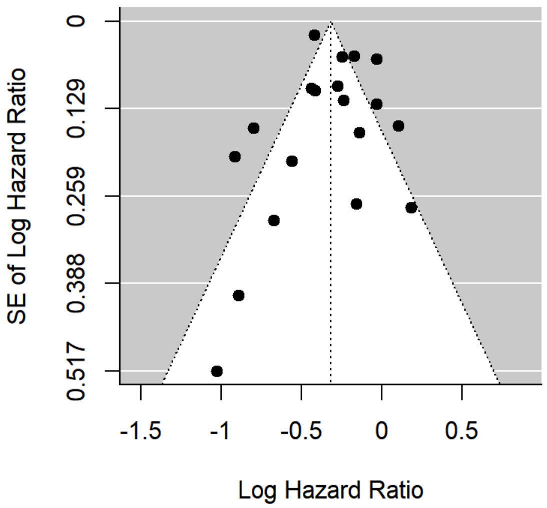

The vertical line represents the pooled HR. The dashed lines represent the pseudo-95% confidence intervals (CI) of the HR. The circles represent HR for each cohort, and the horizontal lines represent standard errors of the HR.

eFigure 3. Funnel Plot of Hazard Ratios (HR) Evaluating the Association Between Statin Use and Overall Mortality Among Men With Androgen-Ablative Therapies for Advanced Prostate Cancer

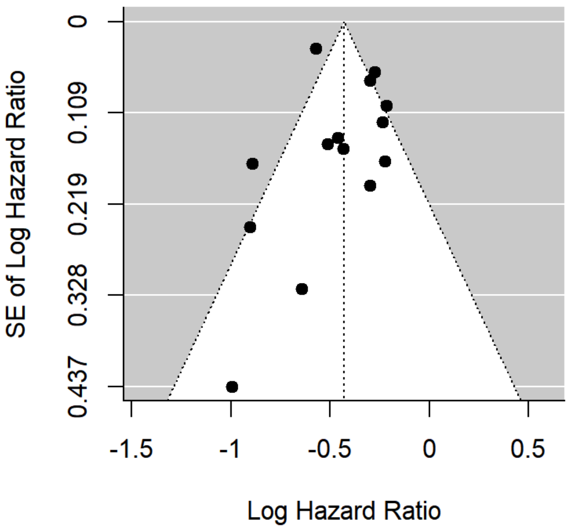

The vertical line represents the pooled HR. The dashed lines represent the pseudo-95% confidence intervals (CI) of the HR. The circles represent HR for each cohort, and the horizontal lines represent standard errors of the HR.

© 2022 Jayalath VH et al. *JAMA Network Open*.

eTable 1. Additional Characteristics of Cohorts Included in Meta-analysis

Study	Initial Therapy	Ascertainment of statin use	PCSM Events	Immortal-time Bias (a)
Anderson-Carter I et al., 2019 (1)	any	prescription database	users: 4,752 (8.9%)<br>non-users: 4,275 (12.6%)	no
Boegemann M et al., 2016 (2)	any	patient interview	-	no
Calais Da Silva Jr F., et al 2015 (3) b	ADT	patient interview	users: 16 (11%)<br>non-users: 32 (17%)	no
Di Lorenzo G et al., 2018 (4)	any	chart review	-	susceptible
Gordon JA et al., 2018 (5)	any	chart review	users: 144 (87%)<br>non-users: 324 (93%)	no
Hamilton RJ et al., 2014 (6) b	any	patient interview	-	no
Hamilton RJ et al., 2020 (7)	RT	patient interview	all: 214 (16%)	no
Henriquez Lopez I et al., 2019 (8) b	-	not reported	-	susceptible
Joentausta RM et al., 2019 (9)	RP	prescription database	-	no
Jung J et al., 2015 (10)	ADT	chart review	all: 132 (77%)	susceptible
Lai et al., 2020 (11) b	any	not reported	-	susceptible
Larsen SB et al., 2017 (12)	ADT alone<br>ADT+RT	prescription database	all (ADT only): 332<br>all (ADT+RT): 72	no
Lorente et al., 2018 (13) b	any	patient interview	-	no
Mikkelsen MK et al., 2017 (14)	ADT	chart review	-	susceptible
Niraula et al., 2011 (15)	any	patient interview	-	no
Peltomaa AI et al., 2021 (16)	any	prescription database	users: 352 (14%)<br>non-users: 482 (26%)	no
Siddiqui S et al., 2019 (17) b	any	chart review	-	susceptible
Tan X et al., 2019 (18)	ADT	prescription database	-	no
Wu SY et al., 2019 (19)	ADT	prescription database	users (M0): 63 (8%)<br>non-users (M0): 95 (8%)<br><br>users (M1): 436 (40%)<br>non-users (M1): 901 (44%)	no

Abbreviations: ADT, androgen-deprivation therapy; NA, not available; RP, radical prostatectomy; RT, radiation therapy.

[a]A cohort was considered susceptible to immortal time bias if: (1) no time-dependent analysis was performed or (2) information regarding timing of statin exposure was not established or available.

[b]Data only available as abstract.

© 2022 Jayalath VH et al. *JAMA Network Open*.

eTable 2. Multivariable Models for Each Study Included in Meta-analyses

Study	Multivariate Model	Covariates by Categories
Anderson-Carter I et al., 2019	age, race, Agent Orange exposure, duration of ADT, CCI, PSA at ADT initiation, diagnosis year, GS	demographic (2)<br>pathological (3)<br>cardiovascular (1)
Boegemann M et al., 2016	chemotherapy status, LN metastases, visceral metastases, bone metastases	demographic (0)<br>pathological (2)<br>cardiovascular (0)
Calais Da Silva Jr F., et al 2015 *	age, mestatatic status	demographic (1)<br>pathological (1)<br>cardiovascular (0)
Di Lorenzo G et al., 2018	age, PSA, ALP, hemoglobin, LDH, neutrophil/lymphocyte ratio, GS, visceral metastases, opiate use, prior chemotherapy, prior enzalutamide	demographic (1)<br>pathological (7)<br>cardiovascular (0)
Gordon JA et al., 2018	age, ALP, neutrophil/lymphocyte ratio, hemoglobin, PSA, CCI, GS, visceral metastases, opiate use	demographic (1)<br>pathological (6)<br>cardiovascular (1)
Hamilton RJ et al., 2014 *	none	demographic (0)<br>pathological (0)<br>cardiovascular (0)
Hamilton RJ et al., 2020	age, time since RT, PSA, prior ADT use	demographic (1)<br>pathological (3)<br>cardiovascular (0)
Henriquez Lopez I et al., 2019 *	hemoglobin, lymphocytes, ALP, pain, opioid use, type of ARAT, RT, ADT, PSA at diagnosis, nadir PSA	demographic (0)<br>pathological (10)<br>cardiovascular (0)
Joentausta RM et al., 2019	age, tumor extent, prior chemotherapy or RT, diabetes, hypertension, CAD, and obesity	demographic (1)<br>pathological (2)<br>cardiovascular (4)
Jung J et al., 2015	diabetes, GS, ADT duration	demographic (0)<br>pathological (2)<br>cardiovascular (1)
Lai et al., 2020 *	GS, primary therapy, prior ADT, metastatic status, and PSA at ADT initiation.	demographic (0)<br>pathological (5)<br>cardiovascular (0)
Larsen SB et al., 2017	age, calendar period, clinical stage, GS, RP, ASA, NSAID, antihypertensive use, CVD drugs, diabetes, COPD, IHD, CHF, CKD, CLD, income, education level, marital status	demographic (4)<br>pathological (3)<br>cardiovascular (10)
Lorente et al., 2018 *	chemotherapy type, baseline PSA, hemoglobin, ALP, visceral metastases, ECOG	demographic (2)<br>pathological (3)<br>cardiovascular (1)
Mikkelsen MK et al., 2017	age, CCI, GS, stage, PSA, metastasis status	demographic (1)<br>pathological (4)<br>cardiovascular (1)
Niraula et al., 2011	treatment group, baseline pain, baseline Karnofsky performance status.	demographic (0)<br>pathological (3)<br>cardiovascular (1)
Peltomaa AI et al., 2021	age, tumor risk group, randomization group, use of antidiabetic and antihypertensive drugs, NSAID, RT	demographic (2)<br>pathological (3)<br>cardiovascular (3)
Siddiqui S et al., 2019 *	age at PSA diagnosis and year of metastasis	demographic (2)<br>pathological (3)<br>cardiovascular (1)
Tan X et al., 2019	age, race, married status, region, median income, education, state buy-in, cancer stage, radiation therapy, surgery, salvage RT, secondary cancer therapy, and CCI	demographic (6)<br>pathological (5)<br>cardiovascular (1)
Wu SY et al., 2019	cancer stage, GS, year of diagnosis, use of metformin, NSAID, ASA	demographic (1)<br>pathological (2)<br>cardiovascular (3)

Abbreviations: ADT – androgen deprivation therapy; CCI – Charlson comorbidity index; PSA – prostate specific antigen; GS – Gleason score; LN – lymph node; ALP – alkaline phosphatase; LDH – lactate dehydrogenase; RT – radiation therapy; ARAT – androgen receptor-axis-targeted the; CAD – coronary artery disease; RP – radical prostatectomy; ASA – acetylsalicylic acid; NSAID – non-steroidal anti-inflammatory drug; CVD – cardiovascular disease; COPD – chronic obstructive pulmonary disease; IHD – ischemic heart disease; CHF – congestive heart failure; CKD – chronic kidney disease; CLD – chronic lung disease; ECOG - Eastern Cooperative Oncology Group performance scale.

* Study data only available in abstract form

© 2022 Jayalath VH et al. *JAMA Network Open*.

eTable 3. Dose-Response Association Between Postdiagnostic Statin Use and Survival Outcomes Among Men on Androgen Ablative Therapies for Advanced Prostate Cancer

Outcome	Study	Dose category	N	Cases	HR (95%CI)
Overall mortality	Peltomaa et al., 2021	DDD1	574	402	0.84 (0.68 to 1.04)
		DDD2	572	207	0.71 (0.62 to 0.82)
		DDD3	575	83	0.61 (0.51 to 0.71)
	Wu et al., 2019	<1 DDD	4852	1991	0.80 (0.72 to 0.89)
		≥1 DDD	4475	1743	0.68 (0.60 to 0.78)
	Anderson-Carter et al., 2019	DDD4	-	-	0.90 (0.82 to 0.99)
	Gordon et al., 2018	Continuous (per mg increase of simvastatin dose equivalent)	123	-	1.00 (0.99 to 1.01)
Prostate cancer-specific mortality	Peltomaa et al., 2021	DDD1	574	160	0.94 (0.69 to 1.29)
		DDD2	572	70	0.67 (0.53 to 0.84)
		DDD3	575	22	0.58 (0.44 to 0.76)
	Wu et al., 2019	<1 DDD	4852	1321	0.83 (0.72 to 0.95)
		≥1 DDD	4475	1170	0.69 (0.58 to 0.81)
	Anderson-Carter et al., 2019	DDD4	-	-	0.74 (0.59 to 0.93)
	Gordon et al., 2018	Continuous (per mg increase of simvastatin dose equivalent)	123	-	1.00 (0.99 to 1.01)

Abbreviations: DDD – defined daily dose; HR – hazard ratio; CI – confidence interval.

© 2022 Jayalath VH et al. *JAMA Network Open*.

eTable 4. Assessment of Study Quality for Cohorts Included in Meta-analysis Using Newcastle-Ottawa Scale

			Cohort Selection				Comparability	Outcome		
Study	Outcome	Score	Representativeness of the Exposed Cohort	Selection of the Non-Exposed Cohort	Ascertainment of Exposure	Demonstration That Outcome of Interest Was Not Present at Start of Study	Comparability of Cohorts on the Basis of the Design or Analysis ($)	Assessment of Outcome	Was Follow-Up Long Enough for Outcomes to Occur (#)	Adequacy of Follow Up of Cohorts
Anderson-Carter I et al., 2019	OM, PCSM	9	1	1	1	1	2	1	1	1
Boegemann M et al., 2016	OM	8	1	1	1	1	1	1	1	1
Calais Da Silva Junior F., et al 2015 *	OM, PCSM	6	1	1	1	1	2	0	0	0
Di Lorenzo G et al., 2018	OM	6	0	1	0	1	2	0	1	1
Gordon JA et al., 2018	OM, PCSM	8	1	1	1	1	2	0	1	1
Hamilton RJ et al., 2014 *	OM	4	0	1	0	1	0	1	1	0
Hamilton RJ et al., 2020	OM, PCSM	8	1	1	0	1	2	1	1	1
Henriquez Lopez I et al., 2019 *	OM	4	0	1	0	1	1	0	1	0
Joentausta RM et al., 2019	PCSM	9	1	1	1	1	2	1	1	1
Jung J et al., 2015	PCSM	8	1	1	1	1	1	1	1	1
Lai et al., 2020 *	OM	3	0	1	0	1	1	0	0	0
Larsen SB et al., 2017	PCSM	9	1	1	1	1	2	1	1	1
Lorente et al., 2018 *	OM	7	1	1	1	1	1	1	1	0
Mikkelsen MK et al., 2017	OM	7	1	1	0	1	2	1	1	0
Niraula et al., 2011	OM	7	1	1	0	1	1	1	1	1
Peltomaa AI et al., 2021	OM, PCSM	9	1	1	1	1	2	1	1	1
Siddiqui S et al., 2019 *	OM	4	0	0	1	1	1	1	0	0
Tan X et al., 2019	OM, PCSM	9	1	1	1	1	2	1	1	1
Wu SY et al., 2019	OM, PCSM	8	1	1	1	1	1	1	1	1

The Newcastle-Ottawa scale was used

Abbreviations: OM – overall mortality; PCSM – prostate cancer-specific mortality

* Study data only available in abstract form

$ Comparability was awarded a maximum of 2 points (1 point for adjusting for age, 1 point for adjusting for baseline PSA , Gleason score, stage or any other baseline risk factor for progression)

Follow-up to >20% of the cohort reaching the outcome of interest was considered sufficiently long enough

© 2022 Jayalath VH et al. *JAMA Network Open*.

eReferences

1. Anderson-Carter I, Posielski N, Liou JI, et al. The impact of statins in combination with androgen deprivation therapy in patients with advanced prostate cancer: a large observational study. Urol Oncol. 2019;37(2):130-137. Medline:30528885 doi:10.1016/j.urolonc.2018.11.017

2. Boegemann M, Schlack K, Fischer AK, et al. Influence of statins on survival outcome in patients with metastatic castration resistant prostate cancer treated with abiraterone acetate. PLoS One. 2016;11(9):e0161959. Medline:27583544 doi:10.1371/journal.pone.0161959

3. Calais Da Silva F, Gonçalves FL, Kliment J, et al. 972 Effects of prior use of statins in a phase 3 study of intermittent versus continuous combined androgen deprivation. Eur Urol Suppl. 2014;13(1):e972. doi:10.1016/S1569-9056(14)60956-8

4. Di Lorenzo G, Sonpavde G, Pond G, et al. Statin use and survival in patients with metastatic castration-resistant prostate cancer treated with abiraterone acetate. Eur Urol Focus. 2018;4(6):874-879. Medline:28753882 doi:10.1016/j.euf.2017.03.015

5. Gordon JA, Buonerba C, Pond G, et al. Statin use and survival in patients with metastatic castration-resistant prostate cancer treated with abiraterone or enzalutamide after docetaxel failure: the international retrospective observational STABEN study. Oncotarget. 2018;9(28):19861-19873. Medline:29731989 doi:10.18632/oncotarget.24888

6. Hamilton RJ, Li J, Naini V, et al. Effect of concomitant medication use on outcomes of treatment and placebo arms of the COU-AA-301 and COU-AA-302 studies of abiraterone acetate (AA) in metastatic castration-resistant prostate cancer (mCRPC). JCO. 2014;32(suppl 15):e16045. doi:10.1200/jco.2014.32.15_suppl.e16045

7. Hamilton RJ, Ding K, Crook JM, et al. The association between statin use and outcomes in patients initiating androgen deprivation therapy. Eur Urol. 2021;79(4):446-452. Medline:33390282 doi:10.1016/j.eururo.2020.12.031

8. Henriquez Lopez I, Gomez J, Torres L, et al. Influence of statins on survival outcome in patients with metastatic castration-resistant prostate cancer treated with androgen receptor inhibitors. Int J Radiat Oncol Biol Physics. 2019;105(1):E278-E279. doi:10.1016/j.ijrobp.2019.06.1908

9. Joentausta RM, Rannikko A, Murtola TJ. Prostate cancer survival among statin users after prostatectomy in a Finnish nationwide cohort. Prostate. 2019;79(6):583-591. Medline:30652328 doi:10.1002/pros.23768

10. Jung J, Lee C, Lee C, et al. Effects of statin use on the response duration to androgen deprivation therapy in metastatic prostate cancer. Korean J Urol. 2015;56(9):630-636. Medline:26366275 doi:10.4111/kju.2015.56.9.630

11. Lai KC, Turknett T, Singh P. Androgen deprivation therapy and statin therapy in prostate cancer patients. J Clin Oncol. 2020;38(suppl 6):205. doi:10.1200/JCO.2020.38.6_suppl.205

12. Larsen SB, Dehlendorff C, Skriver C, et al. Postdiagnosis statin use and mortality in Danish patients with prostate cancer. J Clin Oncol. 2017;35(29):3290-3297. Medline:28806117 doi:10.1200/JCO.2016.71.8981

13. Lorente D, De Velasco Oria GA, Carles D, et al. Statin use and outcome in metastatic castration-resistant prostate cancer (mCRPC) patients (pts) treated in the TROPIC trial. Ann Oncol. 2018;29(suppl 8):viii279-viii280. doi:10.1093/annonc/mdy284.018

14. Mikkelsen MK, Thomsen FB, Berg KD, et al. Associations between statin use and progression in men with prostate cancer treated with primary androgen deprivation therapy. Scand J Urol. 2017;51(6):464-469. Medline:28831860 doi:10.1080/21681805.2017.1362032

15. Niraula S, Pond G, de Wit R, Eisenberger M, Tannock IF, Joshua AM. Influence of concurrent medications on outcomes of men with prostate cancer included in the TAX 327 study. Can Urol Assoc J. 2013;7(1-2):E74-E81. Medline:23671512 doi:10.5489/cuaj.267

© 2022 Jayalath VH et al. *JAMA Network Open*.

16. Peltomaa AI, Raittinen P, Talala K, et al. Prostate cancer prognosis after initiation of androgen deprivation therapy among statin users: a population-based cohort study. Prostate Cancer Prostatic Dis. 2021;24(3):917-924. Medline:33790420 doi:10.1038/s41391-021-00351-2</jrn>

17. Siddiqui S, Durbin-Johnson BP, Yap SA, deVere White RW, Ghosh PM. Abstract 4468: role of statins and PSA nadir after androgen-deprivation therapy in overall survival of patients with metastatic prostate cancer. Cancer Res. 2019;79(suppl 13):4468. doi:10.1158/1538-7445.AM2019-4468.

18. Tan XL, e JY, Lin Y, et al. Individual and joint effects of metformin and statins on mortality among patients with high-risk prostate cancer. Cancer Med. 2020;9(7):2379-2389. Medline:32035002 doi:10.1002/cam4.2862

19. Wu SY, Fang SC, Shih HJ, Wen YC, Shao YJ. Mortality associated with statins in men with advanced prostate cancer treated with androgen deprivation therapy. Eur J Cancer. 2019;112:109-117. Medline:30827745 doi:10.1016/j.ejca.2018.11.032

© 2022 Jayalath VH et al. *JAMA Network Open*.

Appendix 1. Databases and Search Strategy Used

The databases searched included:

Database	Number of articles identified	Search last updated
OVID Medline ALL	784	September 6, 2022
Embase Classic + Embase	2716	September 6, 2022
Cochrane Central Register of Controlled Trials	138	September 6, 2022
Cochrane Database of Systematic Reviews	30	September 6, 2022
Web of Science	1212	September 6, 2022
Total	5190	

© 2022 Jayalath VH et al. *JAMA Network Open*.

Search strategy for each database outlined below:

Ovid MEDLINE(R) ALL to February 10, 2022

#	Searches	Results	Type			
1	exp Prostatic Neoplasms/	138663	Advanced			
2	Prostatic Neoplasms, Castration-Resistant/	4992	Advanced			
3	exp Prostatectomy/	33876	Advanced			
4	Prostate-Specific Antigen/	27229	Advanced			
5	(prostat* adj6 neoplas*).mp,kw.	140981	Advanced			
6	(prostat* adj6 cancer*).mp,kw.	146659	Advanced			
7	(prostat* adj6 tumo?r*).mp,kw.	26005	Advanced			
8	(prostat* adj6 metast*).mp,kw.	22806	Advanced			
9	(prostat* adj6 malignan*).mp,kw.	6849	Advanced			
10	(prostat* adj6 carcin*).mp,kw.	22924	Advanced			
11	(prostat* adj6 adenocarcin*).mp,kw.	10014	Advanced			
12	(prostat* adj6 sarcom*).mp,kw.	807	Advanced			
13	(prostat* adj6 onco*).mp,kw.	3673	Advanced			
14	(prostat* adj6 castrat*).mp,kw.	11625	Advanced			
15	MCRPC.mp,kw.	2219	Advanced			

© 2022 Jayalath VH et al. *JAMA Network Open*.

16	CRPC.mp,kw.	3712	Advanced			
17	prostatectom*.mp,kw.	42200	Advanced			
18	prostat-ectom*.mp,kw.	2	Advanced			
19	(prostate? adj3 resect*).mp,kw.	7323	Advanced			
20	(prostate? adj3 re-sect*).mp,kw.	0	Advanced			
21	(prostate* adj1 specific adj1 antigen?).mp,kw.	40018	Advanced			
22	or/1-21	210615	Advanced			
23	exp Hydroxymethylglutaryl-CoA Reductase Inhibitors/	43804	Advanced			
24	Atorvastatin/	7012	Advanced			
25	Lovastatin/	4665	Advanced			
26	Meglutol/	139	Advanced			
27	Pravastatin/	3494	Advanced			
28	Rosuvastatin Calcium/	2724	Advanced			
29	Simvastatin/	8077	Advanced			
30	statin?.mp,kw.	48083	Advanced			
31	hydroxymethylglutaryl*.mp,kw.	36842	Advanced			
32	hydroxy-methylglutaryl*.mp,kw.	147	Advanced			

© 2022 Jayalath VH et al. *JAMA Network Open*.

33	hydroxymethyl-glutaryl*.mp,kw.	179	Advanced			
34	hydroxy-methyl-glutaryl*.mp,kw.	108	Advanced			
35	(hmg adj1 coenzyme?).mp,kw.	144	Advanced			
36	(hmg adj1 co-enzyme?).mp,kw.	12	Advanced			
37	HMG CoA.mp,kw.	8938	Advanced			
38	HMG Co-A.mp,kw.	158	Advanced			
39	vastatin*.mp,kw.	36	Advanced			
40	atorvastatin*.mp,kw.	10529	Advanced			
41	atorlip*.mp,kw.	1	Advanced			
42	atovarol*.mp,kw.	0	Advanced			
43	cardyl*.mp,kw.	1	Advanced			
44	ci 981.mp,kw.	136	Advanced			
45	ci981.mp,kw.	1	Advanced			
46	glustar*.mp,kw.	0	Advanced			
47	lipibec*.mp,kw.	0	Advanced			
48	lipitor*.mp,kw.	217	Advanced			
49	liprimar*.mp,kw.	4	Advanced			

© 2022 Jayalath VH et al. *JAMA Network Open*.

50	liptonorm*.mp,kw.	2	Advanc ed			
51	lowlipen*.mp,kw.	0	Advanc ed			
52	sortis*.mp,kw.	28	Advanc ed			
53	storvas*.mp,kw.	4	Advanc ed			
54	tahor*.mp,kw.	5	Advanc ed			
55	torvast*.mp,kw.	3	Advanc ed			
56	totalip*.mp,kw.	0	Advanc ed			
57	xarator*.mp,kw.	0	Advanc ed			
58	ym 548.mp,kw.	0	Advanc ed			
59	ym548.mp,kw.	0	Advanc ed			
60	zarator*.mp,kw.	0	Advanc ed			
61	134523-00-5.rn.	0	Advanc ed			
62	134523-03-8.rn.	0	Advanc ed			
63	lovastatin*.mp,kw.	6107	Advanc ed			
64	altocor*.mp,kw.	1	Advanc ed			
65	altoprev*.mp,kw.	8	Advanc ed			
66	artein*.mp,kw.	4	Advanc ed			

© 2022 Jayalath VH et al. *JAMA Network Open*.

67	l 654969.mp,kw.	0	Advanced			
68	lipivas*.mp,kw.	1	Advanced			
69	lovacol*.mp,kw.	0	Advanced			
70	lovahexal*.mp,kw.	0	Advanced			
71	mevacor*.mp,kw.	49	Advanced			
72	mevinacor*.mp,kw.	3	Advanced			
73	mevinolin*.mp,kw.	428	Advanced			
74	"mk 0803".mp,kw.	0	Advanced			
75	mk0803.mp,kw.	0	Advanced			
76	mk 803.mp,kw.	6	Advanced			
77	mk803.mp,kw.	1	Advanced			
78	monacolin*.mp,kw.	321	Advanced			
79	monakolin*.mp,kw.	5	Advanced			
80	msd 803.mp,kw.	1	Advanced			
81	neolipid*.mp,kw.	6	Advanced			
82	6-methylcompactin*.mp,kw.	0	Advanced			
83	75330-75-5.rn.	0	Advanced			

© 2022 Jayalath VH et al. *JAMA Network Open*.

84	meglutol*.mp,kw.	141	Advanced			
85	methylglutaric*.mp,kw.	247	Advanced			
86	methylglutarate*.mp,kw.	56	Advanced			
87	methylpentanedioic*.mp,kw.	1	Advanced			
88	cb 337.mp,kw.	0	Advanced			
89	cb337.mp,kw.	0	Advanced			
90	hydroxymethylglutarate*.mp,kw.	15	Advanced			
91	hydroxymethylglutaric*.mp,kw.	8	Advanced			
92	mevalon*.mp,kw.	7862	Advanced			
93	503-49-1.rn.	0	Advanced			
94	pravastatin*.mp,kw.	5023	Advanced			
95	aplactin*.mp,kw.	0	Advanced			
96	cs 514.mp,kw.	35	Advanced			
97	cs514.mp,kw.	0	Advanced			
98	elisor*.mp,kw.	3	Advanced			
99	epatostantin*.mp,kw.	0	Advanced			
100	eptastatin*.mp,kw.	5	Advanced			

© 2022 Jayalath VH et al. *JAMA Network Open*.

101	lipostat*.mp,kw.	117	Advanced			
102	liprevil*.mp,kw.	0	Advanced			
103	maxudin*.mp,kw.	0	Advanced			
104	mevalotin*.mp,kw.	9	Advanced			
105	minuscol*.mp,kw.	3	Advanced			
106	prareduct*.mp,kw.	0	Advanced			
107	prasterol*.mp,kw.	0	Advanced			
108	pravachol*.mp,kw.	30	Advanced			
109	pravacol*.mp,kw.	1	Advanced			
110	pravalam*.mp,kw.	0	Advanced			
111	pravalich*.mp,kw.	0	Advanced			
112	pravasin*.mp,kw.	0	Advanced			
113	rms 431.mp,kw.	1	Advanced			
114	rms431.mp,kw.	0	Advanced			
115	sanaprav*.mp,kw.	0	Advanced			
116	selectin.tw,kw.	19661	Advanced			
117	selektine*.mp,kw.	1	Advanced			

© 2022 Jayalath VH et al. *JAMA Network Open*.

118	selipran*.mp,kw.	0	Advanced			
119	sq 31000.mp,kw.	1	Advanced			
120	sq31000.mp,kw.	0	Advanced			
121	vasten*.mp,kw.	9	Advanced			
122	81093-37-0.rn.	0	Advanced			
123	81131-70-6.rn.	0	Advanced			
124	rosuvastatin*.mp,kw.	4242	Advanced			
125	crestor*.mp,kw.	74	Advanced			
126	s 4522.mp,kw.	1	Advanced			
127	s4522.mp,kw.	1	Advanced			
128	zd 4522.mp,kw.	0	Advanced			
129	zd4522.mp,kw.	5	Advanced			
130	147098-18-8.rn.	0	Advanced			
131	147098-20-2.rn.	0	Advanced			
132	simvastatin*.mp,kw.	11574	Advanced			
133	belmalip*.mp,kw.	0	Advanced			
134	colemin*.mp,kw.	1	Advanced			

135	denan*.mp,kw.	19	Advanced			
136	epistatin*.mp,kw.	1	Advanced			
137	jabastatina.mp,kw.	0	Advanced			
138	l 644128.mp,kw.	1	Advanced			
139	l644128.mp,kw.	0	Advanced			
140	lipcut*.mp,kw.	0	Advanced			
141	lipex*.mp,kw.	22	Advanced			
142	lipovas*.mp,kw.	1	Advanced			
143	lodales*.mp,kw.	2	Advanced			
144	medipo*.mp,kw.	100	Advanced			
145	mk 733.mp,kw.	41	Advanced			
146	mk733.mp,kw.	1	Advanced			
147	pantok*.mp,kw.	15	Advanced			
148	simva.mp,kw.	63	Advanced			
149	sinvacor*.mp,kw.	1	Advanced			
150	synvinolin*.mp,kw.	20	Advanced			
151	vasilip*.mp,kw.	7	Advanced			

© 2022 Jayalath VH et al. *JAMA Network Open*.

152	zocor*.mp,kw.	123	Advanc ed			
153	79902-63-9.rn.	0	Advanc ed			
154	or/23-153	100782	Advanc ed			
155	22 and 154	798	Advanc ed			
156	exp animals/ not (exp animals/ and exp humans/)	494018 8	Advanc ed			
157	155 not 156	784	Advanc ed			

© 2022 Jayalath VH et al. *JAMA Network Open*.

Embase Classic + Embase to 2022 February 10

#	Searches	Results	Type			
1	exp Prostatic Neoplasms/	271284	Advanced			
2	castration resistant prostate cancer/	15343	Advanced			
3	exp prostatectomy/	66755	Advanced			
4	"patient history of prostatectomy"/	153	Advanced			
5	prostate specific antigen/	63316	Advanced			
6	prostate antigen/	354	Advanced			
7	(prostat* adj6 neoplas*).tw,kw.	7420	Advanced			
8	(prostat* adj6 cancer*).tw,kw.	220573	Advanced			
9	(prostat* adj6 tumo?r*).tw,kw.	34104	Advanced			
10	(prostat* adj6 metast*).tw,kw.	35729	Advanced			
11	(prostat* adj6 malignan*).tw,kw.	9877	Advanced			
12	(prostat* adj6 carcin*).tw,kw.	31121	Advanced			
13	(prostat* adj6 adenocarcin*).tw,kw.	14248	Advanced			
14	(prostat* adj6 sarcom*).tw,kw.	1109	Advanced			
15	(prostat* adj6 onco*).tw,kw.	5229	Advanced			
16	(prostat* adj6 castrat*).tw,kw.	19690	Advanced			
17	MCRPC.tw,kw.	5968	Advanced			
18	CRPC.tw,kw.	7788	Advanced			
19	prostatectom*.tw,kw.	56628	Advanced			
20	prostat-ectom*.tw,kw.	5	Advanced			
21	(prostate? adj3 resect*).tw,kw.	8706	Advanced			
22	(prostate? adj3 re-sect*).tw,kw.	3	Advanced			
23	(prostate* adj1 specific adj1 antigen?).tw,kw.	37053	Advanced			
24	or/1-23	343150	Advanced			
25	exp hydroxymethylglutaryl coenzyme A reductase inhibitor/	170828	Advanced			
26	atorvastatin/	40898	Advanced			
27	mevinolin/	16367	Advanced			
28	3 hydroxy 3 methylglutaric acid/	275	Advanced			
29	pravastatin/	20517	Advanced			
30	rosuvastatin/	16822	Advanced			
31	simvastatin/	39683	Advanced			

© 2022 Jayalath VH et al. *JAMA Network Open*.

32	statin?.tw,kw.	80324	Advanced			
33	hydroxymethylglutaryl*.tw,kw.	2053	Advanced			
34	hydroxy-methylglutaryl*.tw,kw.	186	Advanced			
35	hydroxymethyl-glutaryl*.tw,kw.	230	Advanced			
36	hydroxy-methyl-glutaryl*.tw,kw.	133	Advanced			
37	(hmg adj1 coenzyme?).tw,kw.	177	Advanced			
38	(hmg adj1 co-enzyme?).tw,kw.	12	Advanced			
39	HMG CoA.tw,kw.	11478	Advanced			
40	HMG Co-A.tw,kw.	232	Advanced			
41	vastatin*.tw,kw.	110	Advanced			
42	atorvastatin*.tw,kw.	16106	Advanced			
43	atorlip*.tw,kw.	11	Advanced			
44	atovarol*.tw,kw.	0	Advanced			
45	cardyl*.tw,kw.	8	Advanced			
46	ci 981.tw,kw.	203	Advanced			
47	ci981.tw,kw.	4	Advanced			
48	glustar*.tw,kw.	0	Advanced			
49	lipibec*.tw,kw.	0	Advanced			
50	lipitor*.tw,kw.	2167	Advanced			
51	liprimar*.tw,kw.	17	Advanced			
52	liptonorm*.tw,kw.	2	Advanced			
53	lowlipen*.tw,kw.	0	Advanced			
54	sortis*.tw,kw.	200	Advanced			
55	storvas*.tw,kw.	11	Advanced			
56	tahor*.tw,kw.	70	Advanced			
57	torvast*.tw,kw.	30	Advanced			
58	totalip*.tw,kw.	5	Advanced			
59	xarator*.tw,kw.	1	Advanced			
60	ym 548.tw,kw.	6	Advanced			
61	ym548.tw,kw.	7	Advanced			
62	zarator*.tw,kw.	13	Advanced			
63	134523-00-5.rn.	37474	Advanced			
64	134523-03-8.rn.	37474	Advanced			
65	lovastatin*.tw,kw.	5335	Advanced			

© 2022 Jayalath VH et al. *JAMA Network Open*.

66	altocor*.tw,kw.	46	Advanced			
67	altoprev*.tw,kw.	65	Advanced			
68	artein*.tw,kw.	11	Advanced			
69	l 654969.tw,kw.	19	Advanced			
70	lipivas*.tw,kw.	4	Advanced			
71	lovacol*.tw,kw.	5	Advanced			
72	lovahexal*.tw,kw.	0	Advanced			
73	mevacor*.tw,kw.	820	Advanced			
74	mevinacor*.tw,kw.	100	Advanced			
75	mevinolin*.tw,kw.	525	Advanced			
76	"mk 0803".tw,kw.	2	Advanced			
77	mk0803.tw,kw.	0	Advanced			
78	mk 803.tw,kw.	30	Advanced			
79	mk803.tw,kw.	1	Advanced			
80	monacolin*.tw,kw.	433	Advanced			
81	monakolin*.tw,kw.	15	Advanced			
82	msd 803.tw,kw.	1	Advanced			
83	neolipid*.tw,kw.	9	Advanced			
84	6-methylcompactin*.tw,kw.	0	Advanced			
85	75330-75-5.rn.	15838	Advanced			
86	meglutol*.tw,kw.	4	Advanced			
87	methylglutaric*.tw,kw.	320	Advanced			
88	methylglutarate*.tw,kw.	76	Advanced			
89	methylpentanedioic*.tw,kw.	2	Advanced			
90	cb 337.tw,kw.	2	Advanced			
91	cb337.tw,kw.	0	Advanced			
92	hydroxymethylglutarate*.tw,kw.	15	Advanced			
93	hydroxymethylglutaric*.tw,kw.	11	Advanced			
94	mevalon*.tw,kw.	8252	Advanced			
95	503-49-1.rn.	249	Advanced			
96	pravastatin*.tw,kw.	6084	Advanced			
97	aplactin*.tw,kw.	4	Advanced			
98	cs 514.tw,kw.	80	Advanced			
99	cs514.tw,kw.	0	Advanced			

© 2022 Jayalath VH et al. *JAMA Network Open*.

100	elisor*.tw,kw.	76	Advanced			
101	epatostantin*.tw,kw.	0	Advanced			
102	eptastatin*.tw,kw.	25	Advanced			
103	lipostat*.tw,kw.	235	Advanced			
104	liprevil*.tw,kw.	57	Advanced			
105	maxudin*.tw,kw.	0	Advanced			
106	mevalotin*.tw,kw.	114	Advanced			
107	minuscol*.tw,kw.	0	Advanced			
108	prareduct*.tw,kw.	3	Advanced			
109	prasterol*.tw,kw.	3	Advanced			
110	pravachol*.tw,kw.	666	Advanced			
111	pravacol*.tw,kw.	13	Advanced			
112	pravalam*.tw,kw.	0	Advanced			
113	pravalich*.tw,kw.	0	Advanced			
114	pravasin*.tw,kw.	115	Advanced			
115	rms 431.tw,kw.	1	Advanced			
116	rms431.tw,kw.	0	Advanced			
117	sanaprav*.tw,kw.	5	Advanced			
118	selectin.tw,kw.	27179	Advanced			
119	selektine*.tw,kw.	35	Advanced			
120	selipran*.tw,kw.	42	Advanced			
121	sq 31000.tw,kw.	14	Advanced			
122	sq31000.tw,kw.	0	Advanced			
123	vasten*.tw,kw.	76	Advanced			
124	81093-37-0.rn.	9631	Advanced			
125	81131-70-6.rn.	9631	Advanced			
126	rosuvastatin*.tw,kw.	6976	Advanced			
127	crestor*.tw,kw.	980	Advanced			
128	s 4522.tw,kw.	7	Advanced			
129	s4522.tw,kw.	0	Advanced			
130	zd 4522.tw,kw.	38	Advanced			
131	zd4522.tw,kw.	5	Advanced			
132	147098-18-8.rn.	15151	Advanced			
133	147098-20-2.rn.	15151	Advanced			

© 2022 Jayalath VH et al. *JAMA Network Open*.

134	simvastatin*.tw,kw.	15786	Advanced			
135	belmalip*.tw,kw.	0	Advanced			
136	colemin*.tw,kw.	3	Advanced			
137	denan*.tw,kw.	83	Advanced			
138	epistatin*.tw,kw.	7	Advanced			
139	jabastatina.tw,kw.	0	Advanced			
140	l 644128.tw,kw.	0	Advanced			
141	l644128.tw,kw.	0	Advanced			
142	lipcut*.tw,kw.	0	Advanced			
143	lipex*.tw,kw.	90	Advanced			
144	lipovas*.tw,kw.	30	Advanced			
145	lodales*.tw,kw.	68	Advanced			
146	medipo*.tw,kw.	242	Advanced			
147	mk 733.tw,kw.	84	Advanced			
148	mk733.tw,kw.	1	Advanced			
149	pantok*.tw,kw.	9	Advanced			
150	simva.tw,kw.	148	Advanced			
151	sinvacor*.tw,kw.	12	Advanced			
152	synvinolin*.tw,kw.	38	Advanced			
153	vasilip*.tw,kw.	25	Advanced			
154	zocor*.tw,kw.	2026	Advanced			
155	79902-63-9.rn.	36722	Advanced			
156	or/25-155	227274	Advanced			
157	24 and 156	2728	Advanced			
158	limit 157 to (books or chapter or (book or book series))	12	Advanced			
159	157 not 158	2716	Advanced			

© 2022 Jayalath VH et al. *JAMA Network Open*.

Cochrane Central Register of Controlled Trials to February 10, 2022

#	Searches	Results	Type
1	exp Prostatic Neoplasms/	5997	Advanced
2	Prostatic Neoplasms, Castration-Resistant/	303	Advanced
3	exp Prostatectomy/	1840	Advanced
4	Prostate-Specific Antigen/	1368	Advanced
5	"patient history of prostatectomy"/	0	Advanced
6	prostate antigen/	0	Advanced
7	(prostat* adj6 neoplas*).mp,kw.	6605	Advanced
8	(prostat* adj6 cancer*).mp,kw.	15520	Advanced
9	(prostat* adj6 tumo?r*).mp,kw.	1786	Advanced
10	(prostat* adj6 metast*).mp,kw.	4022	Advanced
11	(prostat* adj6 malignan*).mp,kw.	467	Advanced
12	(prostat* adj6 carcin*).mp,kw.	1245	Advanced
13	(prostat* adj6 adenocarcin*).mp,kw.	1295	Advanced
14	(prostat* adj6 sarcom*).mp,kw.	32	Advanced
15	(prostat* adj6 onco*).mp,kw.	779	Advanced
16	(prostat* adj6 castrat*).mp,kw.	2756	Advanced
17	MCRPC.mp,kw.	1118	Advanced
18	CRPC.mp,kw.	702	Advanced
19	prostatectom*.mp,kw.	4935	Advanced
20	prostat-ectom*.mp,kw.	1	Advanced
21	(prostate? adj3 resect*).mp,kw.	1580	Advanced
22	(prostate? adj3 re-sect*).mp,kw.	0	Advanced
23	(prostate* adj1 specific adj1 antigen?).mp,kw.	4317	Advanced
24	or/1-23	20444	Advanced
25	exp Hydroxymethylglutaryl-CoA Reductase Inhibitors/	5525	Advanced
26	Atorvastatin/	1799	Advanced
27	Lovastatin/	591	Advanced
28	Meglutol/	2	Advanced
29	Pravastatin/	1019	Advanced
30	Rosuvastatin Calcium/	1151	Advanced
31	Simvastatin/	1811	Advanced
32	mevinolin/	591	Advanced

© 2022 Jayalath VH et al. *JAMA Network Open*.

33	3 hydroxy 3 methylglutaric acid/	2	Advanced			
34	statin?.mp,kw.	11107	Advanced			
35	hydroxymethylglutaryl*.mp,kw.	5789	Advanced			
36	hydroxy-methylglutaryl*.mp,kw.	14	Advanced			
37	hydroxymethyl-glutaryl*.mp,kw.	29	Advanced			
38	hydroxy-methyl-glutaryl*.mp,kw.	9	Advanced			
39	(hmg adj1 coenzyme?).mp,kw.	291	Advanced			
40	(hmg adj1 co-enzyme?).mp,kw.	7	Advanced			
41	HMG CoA.mp,kw.	1104	Advanced			
42	HMG Co-A.mp,kw.	56	Advanced			
43	vastatin*.mp,kw.	12	Advanced			
44	atorvastatin*.mp,kw.	5800	Advanced			
45	atorlip*.mp,kw.	1	Advanced			
46	atovarol*.mp,kw.	0	Advanced			
47	cardyl*.mp,kw.	4	Advanced			
48	ci 981.mp,kw.	38	Advanced			
49	ci981.mp,kw.	0	Advanced			
50	glustar*.mp,kw.	0	Advanced			
51	lipibec*.mp,kw.	0	Advanced			
52	lipitor*.mp,kw.	156	Advanced			
53	liprimar*.mp,kw.	0	Advanced			
54	liptonorm*.mp,kw.	0	Advanced			
55	lowlipen*.mp,kw.	0	Advanced			
56	sortis*.mp,kw.	15	Advanced			
57	storvas*.mp,kw.	0	Advanced			
58	tahor*.mp,kw.	2	Advanced			
59	torvast*.mp,kw.	19	Advanced			
60	totalip*.mp,kw.	1	Advanced			
61	xarator*.mp,kw.	0	Advanced			
62	ym 548.mp,kw.	0	Advanced			
63	ym548.mp,kw.	0	Advanced			
64	zarator*.mp,kw.	6	Advanced			
65	lovastatin*.mp,kw.	956	Advanced			
66	altocor*.mp,kw.	0	Advanced			

© 2022 Jayalath VH et al. *JAMA Network Open*.

67	altoprev*.mp,kw.	0	Advanced			
68	artein*.mp,kw.	0	Advanced			
69	l 654969.mp,kw.	0	Advanced			
70	lipivas*.mp,kw.	0	Advanced			
71	lovacol*.mp,kw.	0	Advanced			
72	lovahexal*.mp,kw.	0	Advanced			
73	mevacor*.mp,kw.	15	Advanced			
74	mevinacor*.mp,kw.	2	Advanced			
75	mevinolin*.mp,kw.	146	Advanced			
76	"mk 0803".mp,kw.	0	Advanced			
77	mk0803.mp,kw.	0	Advanced			
78	mk 803.mp,kw.	1	Advanced			
79	mk803.mp,kw.	0	Advanced			
80	monacolin*.mp,kw.	79	Advanced			
81	monakolin*.mp,kw.	6	Advanced			
82	msd 803.mp,kw.	0	Advanced			
83	neolipid*.mp,kw.	0	Advanced			
84	6-methylcompactin*.mp,kw.	0	Advanced			
85	meglutol*.mp,kw.	2	Advanced			
86	methylglutaric*.mp,kw.	7	Advanced			
87	methylglutarate*.mp,kw.	1	Advanced			
88	methylpentanedioic*.mp,kw.	0	Advanced			
89	cb 337.mp,kw.	0	Advanced			
90	cb337.mp,kw.	0	Advanced			
91	hydroxymethylglutarate*.mp,kw.	1	Advanced			
92	hydroxymethylglutaric*.mp,kw.	0	Advanced			
93	mevalon*.mp,kw.	159	Advanced			
94	pravastatin*.mp,kw.	2052	Advanced			
95	aplactin*.mp,kw.	0	Advanced			
96	cs 514.mp,kw.	10	Advanced			
97	cs514.mp,kw.	0	Advanced			
98	elisor*.mp,kw.	2	Advanced			
99	epatostantin*.mp,kw.	0	Advanced			
100	eptastatin*.mp,kw.	1	Advanced			

© 2022 Jayalath VH et al. *JAMA Network Open*.

101	lipostat*.mp,kw.	6	Advanced			
102	liprevil*.mp,kw.	0	Advanced			
103	maxudin*.mp,kw.	0	Advanced			
104	mevalotin*.mp,kw.	6	Advanced			
105	minuscol*.mp,kw.	0	Advanced			
106	prareduct*.mp,kw.	1	Advanced			
107	prasterol*.mp,kw.	0	Advanced			
108	pravachol*.mp,kw.	15	Advanced			
109	pravacol*.mp,kw.	0	Advanced			
110	pravalam*.mp,kw.	0	Advanced			
111	pravalich*.mp,kw.	0	Advanced			
112	pravasin*.mp,kw.	0	Advanced			
113	rms 431.mp,kw.	0	Advanced			
114	rms431.mp,kw.	0	Advanced			
115	sanaprav*.mp,kw.	0	Advanced			
116	selectin.tw,kw.	1981	Advanced			
117	selektine*.mp,kw.	0	Advanced			
118	selipran*.mp,kw.	0	Advanced			
119	sq 31000.mp,kw.	0	Advanced			
120	sq31000.mp,kw.	0	Advanced			
121	vasten*.mp,kw.	2	Advanced			
122	rosuvastatin*.mp,kw.	2753	Advanced			
123	crestor*.mp,kw.	125	Advanced			
124	s 4522.mp,kw.	0	Advanced			
125	s4522.mp,kw.	0	Advanced			
126	zd 4522.mp,kw.	1	Advanced			
127	zd4522.mp,kw.	26	Advanced			
128	simvastatin*.mp,kw.	4072	Advanced			
129	belmalip*.mp,kw.	0	Advanced			
130	colemin*.mp,kw.	1	Advanced			
131	denan*.mp,kw.	0	Advanced			
132	epistatin*.mp,kw.	1	Advanced			
133	jabastatina.mp,kw.	0	Advanced			
134	l 644128.mp,kw.	0	Advanced			

© 2022 Jayalath VH et al. *JAMA Network Open*.

135	l644128.mp,kw.	0	Advanced			
136	lipcut*.mp,kw.	0	Advanced			
137	lipex*.mp,kw.	1	Advanced			
138	lipovas*.mp,kw.	0	Advanced			
139	lodales*.mp,kw.	1	Advanced			
140	medipo*.mp,kw.	51	Advanced			
141	mk 733.mp,kw.	18	Advanced			
142	mk733.mp,kw.	1	Advanced			
143	pantok*.mp,kw.	0	Advanced			
144	simva.mp,kw.	60	Advanced			
145	sinvacor*.mp,kw.	4	Advanced			
146	synvinolin*.mp,kw.	6	Advanced			
147	vasilip*.mp,kw.	0	Advanced			
148	zocor*.mp,kw.	95	Advanced			
149	or/25-148	21891	Advanced			
150	24 and 149	138	Advanced			

© 2022 Jayalath VH et al. *JAMA Network Open*.

Cochrane Database of Systematic Reviews to February 10, 2022

#	Searches	Results	Type			
1	(prostat* adj6 neoplas*).mp,kw.	43	Advanced			
2	(prostat* adj6 cancer*).mp,kw.	204	Advanced			
3	(prostat* adj6 tumo?r*).mp,kw.	33	Advanced			
4	(prostat* adj6 metast*).mp,kw.	30	Advanced			
5	(prostat* adj6 malignan*).mp,kw.	41	Advanced			
6	(prostat* adj6 carcin*).mp,kw.	28	Advanced			
7	(prostat* adj6 adenocarcin*).mp,kw.	13	Advanced			
8	(prostat* adj6 sarcom*).mp,kw.	0	Advanced			
9	(prostat* adj6 onco*).mp,kw.	5	Advanced			
10	(prostat* adj6 castrat*).mp,kw.	12	Advanced			
11	MCRPC.mp,kw.	1	Advanced			
12	CRPC.mp,kw.	0	Advanced			
13	prostatectom*.mp,kw.	69	Advanced			
14	prostat-ectom*.mp,kw.	0	Advanced			
15	(prostate? adj3 resect*).mp,kw.	47	Advanced			
16	(prostate? adj3 re-sect*).mp,kw.	0	Advanced			
17	(prostate* adj1 specific adj1 antigen?).mp,kw.	61	Advanced			
18	or/1-17	271	Advanced			
19	statin?.mp,kw.	808	Advanced			
20	hydroxymethylglutaryl*.mp,kw.	47	Advanced			
21	hydroxy-methylglutaryl*.mp,kw.	2	Advanced			
22	hydroxymethyl-glutaryl*.mp,kw.	1	Advanced			
23	hydroxy-methyl-glutaryl*.mp,kw.	0	Advanced			
24	(hmg adj1 coenzyme?).mp,kw.	4	Advanced			
25	(hmg adj1 co-enzyme?).mp,kw.	2	Advanced			
26	HMG CoA.mp,kw.	48	Advanced			
27	HMG Co-A.mp,kw.	9	Advanced			
28	vastatin*.mp,kw.	1	Advanced			
29	atorvastatin*.mp,kw.	68	Advanced			
30	atorlip*.mp,kw.	2	Advanced			
31	atovarol*.mp,kw.	2	Advanced			
32	cardyl*.mp,kw.	2	Advanced			

© 2022 Jayalath VH et al. *JAMA Network Open*.

33	ci 981.mp,kw.	15	Advanced			
34	ci981.mp,kw.	5	Advanced			
35	glustar*.mp,kw.	2	Advanced			
36	lipibec*.mp,kw.	4	Advanced			
37	lipitor*.mp,kw.	11	Advanced			
38	liprimar*.mp,kw.	1	Advanced			
39	liptonorm*.mp,kw.	2	Advanced			
40	lowlipen*.mp,kw.	2	Advanced			
41	sortis*.mp,kw.	5	Advanced			
42	storvas*.mp,kw.	2	Advanced			
43	tahor*.mp,kw.	5	Advanced			
44	torvast*.mp,kw.	5	Advanced			
45	totalip*.mp,kw.	2	Advanced			
46	xarator*.mp,kw.	2	Advanced			
47	ym 548.mp,kw.	5	Advanced			
48	ym548.mp,kw.	5	Advanced			
49	zarator*.mp,kw.	5	Advanced			
50	lovastatin*.mp,kw.	45	Advanced			
51	altocor*.mp,kw.	4	Advanced			
52	altoprev*.mp,kw.	3	Advanced			
53	artein*.mp,kw.	3	Advanced			
54	l 654969.mp,kw.	3	Advanced			
55	lipivas*.mp,kw.	2	Advanced			
56	lovacol*.mp,kw.	4	Advanced			
57	lovahexal*.mp,kw.	0	Advanced			
58	mevacor*.mp,kw.	7	Advanced			
59	mevinacor*.mp,kw.	5	Advanced			
60	mevinolin*.mp,kw.	11	Advanced			
61	"mk 0803".mp,kw.	2	Advanced			
62	mk0803.mp,kw.	3	Advanced			
63	mk 803.mp,kw.	4	Advanced			
64	mk803.mp,kw.	4	Advanced			
65	monacolin*.mp,kw.	6	Advanced			
66	monakolin*.mp,kw.	1	Advanced			

© 2022 Jayalath VH et al. *JAMA Network Open*.

67	msd 803.mp,kw.	3	Advanced			
68	neolipid*.mp,kw.	4	Advanced			
69	6-methylcompactin*.mp,kw.	1	Advanced			
70	meglutol*.mp,kw.	12	Advanced			
71	methylglutaric*.mp,kw.	4	Advanced			
72	methylglutarate*.mp,kw.	3	Advanced			
73	methylpentanedioic*.mp,kw.	3	Advanced			
74	cb 337.mp,kw.	0	Advanced			
75	cb337.mp,kw.	0	Advanced			
76	hydroxymethylglutarate*.mp,kw.	0	Advanced			
77	hydroxymethylglutaric*.mp,kw.	0	Advanced			
78	mevalon*.mp,kw.	15	Advanced			
79	pravastatin*.mp,kw.	46	Advanced			
80	aplactin*.mp,kw.	2	Advanced			
81	cs 514.mp,kw.	5	Advanced			
82	cs514.mp,kw.	5	Advanced			
83	elisor*.mp,kw.	5	Advanced			
84	epatostantin*.mp,kw.	4	Advanced			
85	eptastatin*.mp,kw.	5	Advanced			
86	lipostat*.mp,kw.	9	Advanced			
87	liprevil*.mp,kw.	3	Advanced			
88	maxudin*.mp,kw.	1	Advanced			
89	mevalotin*.mp,kw.	6	Advanced			
90	minuscol*.mp,kw.	1	Advanced			
91	prareduct*.mp,kw.	4	Advanced			
92	prasterol*.mp,kw.	1	Advanced			
93	pravachol*.mp,kw.	9	Advanced			
94	pravacol*.mp,kw.	5	Advanced			
95	pravalam*.mp,kw.	1	Advanced			
96	pravalich*.mp,kw.	1	Advanced			
97	pravasin*.mp,kw.	6	Advanced			
98	rms 431.mp,kw.	4	Advanced			
99	rms431.mp,kw.	4	Advanced			
100	sanaprav*.mp,kw.	5	Advanced			

101	selectin.tw,kw.	23	Advanced			
102	selektine*.mp,kw.	5	Advanced			
103	selipran*.mp,kw.	3	Advanced			
104	sq 31000.mp,kw.	5	Advanced			
105	sq31000.mp,kw.	5	Advanced			
106	vasten*.mp,kw.	6	Advanced			
107	rosuvastatin*.mp,kw.	44	Advanced			
108	crestor*.mp,kw.	8	Advanced			
109	s 4522.mp,kw.	5	Advanced			
110	s4522.mp,kw.	5	Advanced			
111	zd 4522.mp,kw.	5	Advanced			
112	zd4522.mp,kw.	5	Advanced			
113	simvastatin*.mp,kw.	80	Advanced			
114	belmalip*.mp,kw.	0	Advanced			
115	colemin*.mp,kw.	1	Advanced			
116	denan*.mp,kw.	4	Advanced			
117	epistatin*.mp,kw.	3	Advanced			
118	jabastatina.mp,kw.	0	Advanced			
119	l 644128.mp,kw.	3	Advanced			
120	l644128.mp,kw.	3	Advanced			
121	lipcut*.mp,kw.	1	Advanced			
122	lipex*.mp,kw.	5	Advanced			
123	lipovas*.mp,kw.	5	Advanced			
124	lodales*.mp,kw.	4	Advanced			
125	medipo*.mp,kw.	4	Advanced			
126	mk 733.mp,kw.	4	Advanced			
127	mk733.mp,kw.	4	Advanced			
128	pantok*.mp,kw.	1	Advanced			
129	simva.mp,kw.	0	Advanced			
130	sinvacor*.mp,kw.	4	Advanced			
131	synvinolin*.mp,kw.	2	Advanced			
132	vasilip*.mp,kw.	1	Advanced			
133	zocor*.mp,kw.	9	Advanced			
134	or/19-133	870	Advanced			

© 2022 Jayalath VH et al. *JAMA Network Open*.

135	18 and 134	30	Advanced			

© 2022 Jayalath VH et al. *JAMA Network Open*.

Web of Science Core Collection from Clarivate Analytics to February 10, 2022

#1 278147
TS=((prostat* NEAR/6 neoplas*) OR (prostat* NEAR/6 cancer*) OR (prostat* NEAR/6 tumo?r*) OR (prostat* NEAR/6 metast*) OR (prostat* NEAR/6 malignan*) OR (prostat* NEAR/6 carcin*) OR (prostat* NEAR/6 adenocarcin*) OR (prostat* NEAR/6 sarcom*) OR (prostat* NEAR/6 onco*) OR (prostat* NEAR/6 castrat*) OR MCRPC OR CRPC OR prostatectom* OR prostat-ectom* OR (prostate? NEAR/3 resect*) OR (prostate? NEAR/3 re-sect*) OR (prostate* NEAR/1 specific NEAR/1 antigen?))

All Editions

#2 133232
TS=(statin? OR hydroxymethylglutaryl* OR hydroxy-methylglutaryl* OR hydroxymethyl-glutaryl* OR hydroxy-methyl-glutaryl* OR (hmg NEAR/1 coenzyme?) OR (hmg NEAR/1 co-enzyme?) OR HMG CoA OR HMG Co-A OR vastatin* OR atorvastatin* OR atorlip* OR atovarol* OR cardyl* OR ci 981 OR ci981 OR glustar* OR lipibec* OR lipitor* OR liprimar* OR liptonorm* OR lowlipen* OR sortis* OR storvas* OR tahor* OR torvast* OR totalip* OR xarator* OR ym 548 OR ym548 OR zarator* OR lovastatin* OR altocor* OR altoprev* OR artein* OR l 654969 OR lipivas* OR lovacol* OR lovahexal* OR mevacor* OR mevinacor* OR mevinolin* OR "mk 0803" OR mk0803 OR mk 803 OR mk803 OR monacolin* OR monakolin* OR msd 803 OR neolipid* OR 6-methylcompactin* OR meglutol* OR methylglutaric* OR methylglutarate* OR methylpentanedioic* OR cb 337 OR cb337 OR hydroxymethylglutarate* OR hydroxymethylglutaric* OR mevalon* OR pravastatin* OR
aplactin* OR cs 514 OR cs514 OR elisor* OR epatostantin* OR eptastatin* OR lipostat* OR liprevil* OR maxudin* OR mevalotin* OR minuscol* OR prareduct* OR prasterol* OR pravachol* OR pravacol* OR pravalam* OR pravalich* OR pravasin* OR rms 431 OR rms431 OR sanaprav* OR selectin OR selektine* OR selipran* OR sq 31000 OR sq31000 OR vasten* OR rosuvastatin* OR crestor* OR s 4522 OR s4522 OR zd 4522 OR zd4522 OR simvastatin* OR belmalip* OR colemin* OR denan* OR epistatin* OR jabastatina OR l 644128 OR l644128 OR lipcut* OR lipex* OR lipovas* OR lodales* OR medipo* OR mk 733 OR mk733 OR pantok* OR simva OR sinvacor* OR synvinolin* OR vasilip* OR zocor*)

All Editions

#3 1212
#1 AND #2

© 2022 Jayalath VH et al. *JAMA Network Open*.

eAppendix 2. GRADE Assessment of Quality of Evidence

Certainty assessment							№ of patients		Effect		Certainty	Importance
№ of studies	Study design	Risk of bias	Inconsistency	Indirectness	Imprecision	Other considerations	Statin use	no statin use	Relative (95% CI)	Absolute (95% CI)		
Overall Survival												
19	observational studies	not serious[a]	serious[b]	not serious	not serious	dose response gradient[c]	-/61630	-/46882	**HR 0.73** (0.66 to 0.82)	**-- per 1,000** (from -- to --)[d]	⨁⨁◯◯ Low	IMPORTANT
Prostate Cancer-Specific Mortality												
14	observational studies	not serious[e]	serious[b]	not serious	not serious	dose response gradient[c]	-/64511	-/51101	**HR 0.65** (0.58 to 0.73)	**-- per 1,000** (from -- to --)[d]	⨁⨁◯◯ Low	IMPORTANT

CI: confidence interval; **HR:** hazard Ratio

Explanations

a. 12 of 19 studies were considered high quality by the Newcastle-Ottawa Scale. Removing reports published only as abstracts nor subgroup assessment by low vs high quality study changed the overall association.

b. While the vast majority of studies supported a protective association with statins, there was significant inter-study heterogeneity that was not explained by subgroup or sensitivity analyses. However, it should be noted that the inconsistency was between large and small protective associations, suggesting consistency in a beneficial relationship.

c. Three studies (Peltomaa et al., 2021, Wu et al., 2019, and Anderson-Carter et al., 2019) identified significant dose-response associations by defined daily dose (DDD); one study (Di Lorenzo et al., 2018) evaluated a dose-response per milligram of simvastatin-equivalent statin dose and did not find a significant relationship.

d. Absolute estimates were not calculated as event rates were not accurately available for all included studies.

e. 13 of 14 studies were considered high quality by the Newcastle-Ottawa Scale. Removing reports published only as abstracts nor subgroup assessment by low vs high quality study changed the overall association.

© 2022 Jayalath VH et al. *JAMA Network Open*.
