# Supplementary material for: Statin Use and Survival Among Men Receiving Androgen-Ablative Therapies for Advanced Prostate Cancer: A Systematic Review and Meta-analysis
Source: JAMA Netw Open. 2022 Nov 30;5(11):e2242676. doi: 10.1001/jamanetworkopen.2022.42676 (PMC9713611; doi:10.1001/jamanetworkopen.2022.42676)
Supplement: Supplement 2. — Data Sharing Statement [file jamanetwopen-e2242676-s002.pdf]

Data Sharing Statement

Jayalath. Statin Use and Survival Among Men Receiving Androgen-Ablative Therapies for Advanced Prostate Cancer: A Systematic Review and Meta-analysis. *JAMA Netw Open*. Published November 30, 2022. doi:10.1001/jamanetworkopen.2022.42676

Data
Data available: No

Additional Information
Explanation for why data not available: All data used in this study are publicly available; we did not use any individual patient data for this meta-analysis
